# Evaluating the potential of bioacoustics in avian migration research by citizen science and weather radar observations

**DOI:** 10.1371/journal.pone.0299463

**Published:** 2024-03-08

**Authors:** Nadja Weisshaupt, Juha Saari, Jarmo Koistinen

**Affiliations:** 1 Space Research and Observation Technologies, Finnish Meteorological Institute, Helsinki, Finland; 2 Independent Researcher, Helsinki, Finland; Universidade de Aveiro, PORTUGAL

## Abstract

The study of nocturnal bird migration brings observational challenges because of reduced visibility and observability of birds at night. Remote sensing tools, especially radars, have long been the preferred choice of scientists to study nocturnal migrations. A major downside of these remote sensing tools is the lack of species-level information. With technological advances in recent decades and with improved accessibility and affordability of acoustic tools, sound recordings have steeply increased in popularity. In Europe, there is no exhaustive qualitative and quantitative evaluation of the content of such acoustic databases and therefore the value for migration science and migration-related applications, such as bird collision hazard assessments, is mostly unknown. In the present work we compared migration schedules estimated from citizen science data with quantitative temporal occurrence of species in four years of acoustic recordings. Furthermore, we contrasted acoustic recordings with citizen science observations and weather radar data from one spring and one autumn season to assess the qualitative and quantitative yield of acoustic recordings for migration-related research and applications. Migration intensity estimated from weather radar data correlated best at low levels with acoustic records including all species in spring while in autumn passerine species showed stronger correlation than the entire species composition. Our findings identify a minor number of species whose call records may be eligible for applications derived from acoustics. Especially the highly vocal species Song thrush and Redwing showed relatively good correlations with radar and citizen science migration schedules. Most long-distance passerine migrants and many other migrants were not captured by acoustics and an estimated seasonal average of about 50% of nocturnally migrating passerine populations remained undetected. Overall, the ability of acoustic records to act as a proxy of overall migration dynamics is highly dependent on the migration period and species involved.

## Introduction

In times of global change, the policy-driven quest for sustainable solutions also challenges scientists collecting relevant field data to advise of potential risks for the environment through new innovations. Bird collisions with anthropogenic structures are a well-known problem both in aviation and in the wind energy sector, particularly during migration seasons (e.g. [1, 2]). With the rising demand for renewable energy sources and the development of air traffic, pressure for effective standardized monitoring in various settings is increasing [3, 4]. Especially at night, the study of bird movements implies observational challenges because of the reduced visibility and observability of birds. Remote sensing tools, such as radars or thermal imaging, or moonwatching (e.g. [5]) have long been the preferred choice of scientists to study nocturnal migrations. A downside of these remote sensing systems is the lack of species-specific information, which would be indispensable for applications in biodiversity studies. This species dilemma can be partly remedied by citizen science (CS) observations from bird portals [6]. However, such observations are mostly obtained in daylight and not simultaneously at night. With technological advances in recent decades and with improved accessibility and affordability of acoustic tools, sound recordings have steeply increased in popularity [7]. In recent years, the study of nocturnal calls has strongly developed on the American continent (e.g. [8]). [9] even consider acoustic monitoring superior to radars as their study provides both quantitative and taxonomical information for several species. In Europe, there have been several acoustic studies by human ear already many decades ago, e.g. [10–14]. All of them report strong presence of thrush calls amongst some other species. Despite these early works acoustic observations have remained rather in the amateur sector of migration ornithology. In particular, bird sound recordings are gathered on online portals, such as The Sound Approach (https://soundapproach.co.uk), Xeno-canto (https://xeno-canto.org) or Trektellen [15], and anyone can contribute their samples. Much of recent research is addressing mainly methodological issues and is increasingly exploring automated processing methods (e.g. [16, 17]. While there seems to be a certain potential in ground-based monitoring (e.g. [18]), the potential of acoustic data for nocturnal bird migration monitoring is still largely unexplored in Europe [19]. [20] outline the usefulness of acoustics in nocturnal observations, but there is no comparison or validation available with methods established for nocturnal migration studies. There is thus a serious methodological knowledge gap as to the detection capacity and species coverage of acoustic methods and thus the informational value of the data for migration research. Such an evaluation is pivotal to inform potential applications and integration of acoustics e.g. in environmental impact assessments and bird collision mitigation measures related to bird migration. It is essential to contrast acoustic species composition with other quantitative and qualitative data sources to untap any additional value acoustics may (or may not) deliver to science. Such an evaluation requires the combination of various data sources from established methodologies. CS has been shown to provide migration schedules for a wide range of species which facilitates estimating expected species compositions for both diurnal and nocturnal migrants at various spatiotemporal scales [21]. Population data from standardized bird surveys together with data on offspring production and survival rates allow estimating expected sizes of migrating population and thus proportional bird compositions during migration seasons. Finally, radars, as probably the most established tools for nocturnal bird migration studies, provide an overview of bird fluxes from local to large scale [22]. By combining these various sources of data, it is possible to obtain an approximation of both biomass and expected species composition to evaluate the potential of acoustic data for migration research.

In this study, we combine weather radar data, population estimates from standardized bird censuses and bird migration schedules from CS observations to assess the informative value of acoustic species compositions and quantitative estimates of nocturnal bird migration to reflect overall migration fluxes. We furthermore evaluate the potential of acoustic data as taxonomical support in weather radar analyses. We hypothesize that acoustic data can deliver temporal occurrence for species with sufficient records if accumulated over multiple years as well as presence-only data in single-year use. However, numbers of recorded migrants may not be representative of overall migration dynamics as measured by radars since silent migrants would remain undetected.

## Material & methods

### Study data

Acoustic data from 1 March to 31 October 2019–2022 (four years) were collected in the Pihlajamäki district of Helsinki (60.2357 N, 25.0057 E). Data are available on Trektellen https://www.trektellen.nl/site/info/2204. Bird calls were recorded from sunset to sunrise using a Telinga PRO-X parabolic microphone, sometimes combined with an Røde NTG8 microphone pointing in a slightly different direction to improve coverage. Species were identified acoustically and visually by checking the sonograms in Audacity v. 2.2 software [23]. We included only individuals that could be unequivocally identified to species level. The proportion of unidentified individuals in the entire dataset was about 1–2% and of individuals identified to genus or family level about 15%. In this study, the acoustic counts refer to the number of bird individuals considering that species can utter multiple calls while passing. Individual birds were identified by sonogram call sequence structure, which differs in approaching, peak and leaving stages of birds’ flight trajectory relative to the microphone, i.e. the call sequence becomes clearer and stronger, when the bird is approaching, and fainter, when it is leaving. Individuals flying together simultaneously were separated based on flight call series in sonogram (comparable to tracks of individuals in the snow). The estimation of flock size from calls is a conservative approximation based on expert field knowledge from day observations and related call frequency (i.e. a comparison of flock size and associated call numbers), simultaneous thermal imaging at night and overflight time considering that nocturnal and diurnal call frequency may differ. The number of calling individuals were then summed per species per night.

10 years of CS observations were obtained for the 114 most abundant bird species in Finland from the online bird portal *Tiira* maintained by BirdLife Finland (S1 Table). These species make up the bulk of migratory bird populations in Finland. Spring and autumn migration schedules were estimated following [21]. For this study, migration schedules for zone 1 in South Finland were used. Despite the larger catchment area of this zone compared to the range of the recording device and radar, the migration schedules apply to the entire area. For the same species and area, population data were obtained from Finnish bird censuses [24, 25]. Migratory population proportions passing through Finland were added to the Finnish breeding populations considering the most likely recruitment areas, especially in Russia, and species distributions as well as each species’ preferred migration direction estimated from Finnish ringing recaptures [26, 27]. Offspring numbers were added to autumn migration populations estimated from species-specific survival rates and offspring numbers from Finnish literature, if available, or from populations as close to the Finnish ones as possible, mainly from [28], complemented by some more recent accounts in [29, 30]. As survival rates are reported from various juvenile stages (post-fledging up to first winter survival), we adapted survival rates based on general findings on survival variability during early life stages (e.g. [31, 32]) and number of clutches in Finland [33] to estimate rates suitable for summer/autumn populations before birds’ departure. Furthermore, for partial migrants the average sedentary population proportion was estimated based on winter bird censuses [34]. Final migrant population sizes are summarized in S1 Table.

Radar data were obtained from the polarimetric C-band weather radar in Vihti (60.5562 N, 24.4956 E) about 45 km from the audio recording site in Helsinki, Finland. Data consisted of hourly volume scans (one volume scan per hour) between sunset and sunrise from 1 April to 31 May and 1 August to 31 October 2022. Bird echoes were identified by means of the Bayesian classification methodology by [35] including radar scans in a radius of 5–35 km around the radar. The radar samples (bins) classified as birds with a probability of more than 50% were then further used to estimate average bird densities sensu [36] per 200-meter height layers up to four kilometers. The threshold of 50% was set as a conservative threshold based on visual inspection of bird probabilities which typically range between 80–100%. Bird probabilities of less than 50% are at an increasing risk of being false positives. Bird densities in layers were then summed vertically and hourly between sunset and sunrise to obtain accumulated nightly bird densities. We opted for bird densities in place of migration traffic rates typically used in migration studies [37] because we wanted to capture also non-passerines forming heterogenous migration phenomena such as streams of waterfowl migrations [6], whose species might be part of the audio recordings. Such spatially confined migrations do not work with VAD approaches needed to calculate flight speeds for migration traffic rates from weather radar data [38, 39].

### Data analysis

#### Citizen science observations vs. acoustic data

For the analyses we included species for which both the CS migration schedules and acoustic records were available (see S1 Table), so overall 59 species.

We compared the quantities and temporal occurrence of calling individuals of all species with a minimum of 10 call nights between 1 March and 31 October stacked for all four years with the CS migration schedules. The aim was to check if the acoustic data could be indicative of a species’ migration phenology. For the quantitative part, we performed Spearman’s rank correlation analyses to account for non-normality in the data for the two seasons spring (1 Mar to 31 May) and autumn (1 Jun– 31 Oct) separately. To compare migration phenology between acoustics and CS data, we calculated 5%, 50% and 95% percentiles of the acoustic data for the two seasons spring (1 Mar to 31 May) and autumn (1 Jun– 31 Oct) separately and contrasted them with the CS migration timing for zone 1 in [21]. For visualization purposes we allocated the daily data in respective pentads from 2 March to 31 Oct.

For further quantitative assessment of the acoustic data, we combined the population estimates with the expected daily proportions for each species according to the CS migration schedules, which represents an approximation of the expected species composition in respective proportions.

#### Radar observations vs. acoustic data

For the comparison with radar data, we checked whether the number of calling birds of all recorded species correlated with bird densities measured by the weather radar in Vihti in spring and autumn 2022. We calculated Spearman’s rank correlation coefficients and fitted linear regression models for the entire bird species composition and for passerine species only to test for any effect of potential quantitative dominance of passerines in radar data. We also extracted bird densities for the lowest height layer below 200 m a.g.l. to check whether bird densities in this scan volume would better fit with acoustic counts in the microphone range. To obtain an overview of birds’ height distributions which may contribute to observed correlation patterns between the acoustic data and the lowest layer, we generated height profiles of mean bird proportions in 200-m layers from 0 to 4 kilometers per season. Only nights when both data sources were available were included, i.e. 55 nights in spring and 87 nights in autumn. Correlation analyses were performed for the spring season between 1 April and 31 May and for the autumn season from 1 August to 31 October 2022. Furthermore, spring and autumn season were subdivided into partial seasons from 1–30 April and 1–31 May, and 1 August to 9 Sept and from 10 Sept to 30 Oct. This separation was intended to detect potential impacts of shifts in species composition, i.e. May, August and early Sept are dominated by long-distance migrants and April and mid-September onwards by short-distance migrants. Species with at least 100 calling individuals (i.e. “dominating” species) distributed across 10 nights in each spring and autumn 2022 were included to determine how well single species reflect nocturnal activity observed in radar data. We calculated Spearman’s rank correlation coefficients and fitted linear regression models to each of these species between the 5^th^ and 95^th^ percentile of their species-specific migration period identified by CS data in [21].

Analyses were performed in Python 3 [40] and R [41].

## Results

### Citizen science observations vs. acoustic data

The acoustic dataset contained calls of 81 species (S1 Table). CS migration schedules were available for 59 of those species. Of those, 27 species yielded less than 10 call nights in the four-year acoustic dataset and were therefore excluded. The final species selection in the comparison between CS and acoustic data consisted of 32 species. Five species had more than 1000 calling individuals in the four-year period: Tree pipit (*Anthus trivialis)*, Barnacle goose (*Branta leucopsis*), Common scoter (*Melanitta nigra*), Redwing (*Turdus iliacus*) and Song thrush (*Turdus philomelos*). There were no records of *Sylvia spp*., *Acrocephalus spp*. or *Phylloscopus spp*. in the recordings. The only records of insectivorous long-distance migrants, which typically represent the bulk of nocturnal passerine migrants in May, August and early September in Finland [21], were from the Common redstart (*Phoenicurus phoenicurus*) (2 calling individuals), Pied flycatcher (*Ficedula hypoleuca*) (23 calling individuals), Spotted flycatcher (72 calling individuals) and the partially nocturnal migrant Tree pipit (1082 calling individuals).

**Fig 1:**
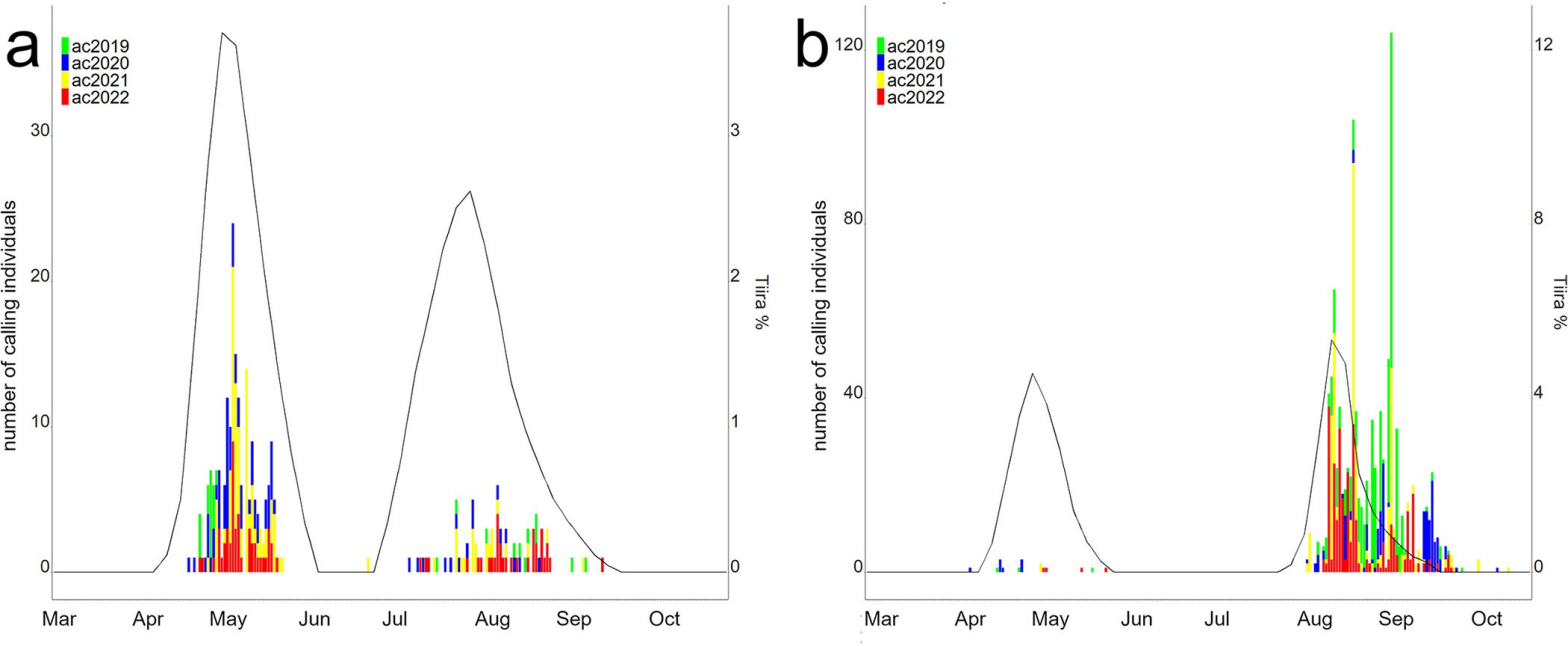
Two examples of migration phenologies based on acoustic and citizen science data. Acoustic counts for the years 2019–2022 (stacked color bars) and citizen science migration phenologies (black line) from the bird portal *Tiira* for (a) the Common sandpiper (*A*. *hypoleucos*) and (b) the Tree pipit (*A*. *trivialis*).

The yield of acoustic records and informational content varied greatly with species. In the data stacked over four years, some species exhibited clear patterns of migration phenology (onset, peak and end), while for others data was very scarce (S1A–S1Af Fig include species with at least 10 nights of acoustic observations). As examples, we show the migration phenology of the Common sandpiper (*Actitis hypoleucos*) and the Tree pipit in Fig 1A and 1B.

In the correlation analyses between the CS and the pooled four-year acoustic data, the only species with a high R-squared of more than 0.5 were the Common sandpiper in spring (R^2^ = 0.59, Spearman’s rho = 0.85, F = 127.46, P < 0.001, n = 32) and the Song thrush in autumn (R^2^ = 0.55, Spearman’s rho = 0.88, F = 148.47, P < 0.001, n = 84). Six species (Eurasian teal (*Anas crecca*), Mallard (*Anas platyrhynchos*), Long-tailed duck (*Clangula hyemalis*), European golden plover (*Pluvialis apricaria*), Blackbird (*Turdus merula*) and Song thrush) had an R^2^ between 0.2 and 0.5 in spring and 14 species (Common sandpiper, Tree pipit, Barnacle goose, Yellowhammer (*Emberiza citrinella*), European robin (*Erithacus rubecula*), Pied flycatcher (*Ficedula hypoleuca*), Spotted flycatcher (*Muscicapa striata*), Dunnock (*Prunella modularis*), Goldcrest (*Regulus regulus*), Wood sandpiper (*Tringa glareola*), Green sandpiper (*Tringa ochropus*), Blackbird, Redwing and Fieldfare (*Turdus pilaris*)) in autumn (see S2 Table). R^2^<0.1 were found in five species (Eurasian skylark (*Alauda arvensis*), Mallard, Eurasian siskin (*Carduelis spinus*), Common snipe (*Gallinago gallinago*) and European golden plover) in autumn and none in spring. Overall, correlations between single-year acoustic and CS migration schedules were typically weaker than multi-year data (not shown).

**Fig 2:**
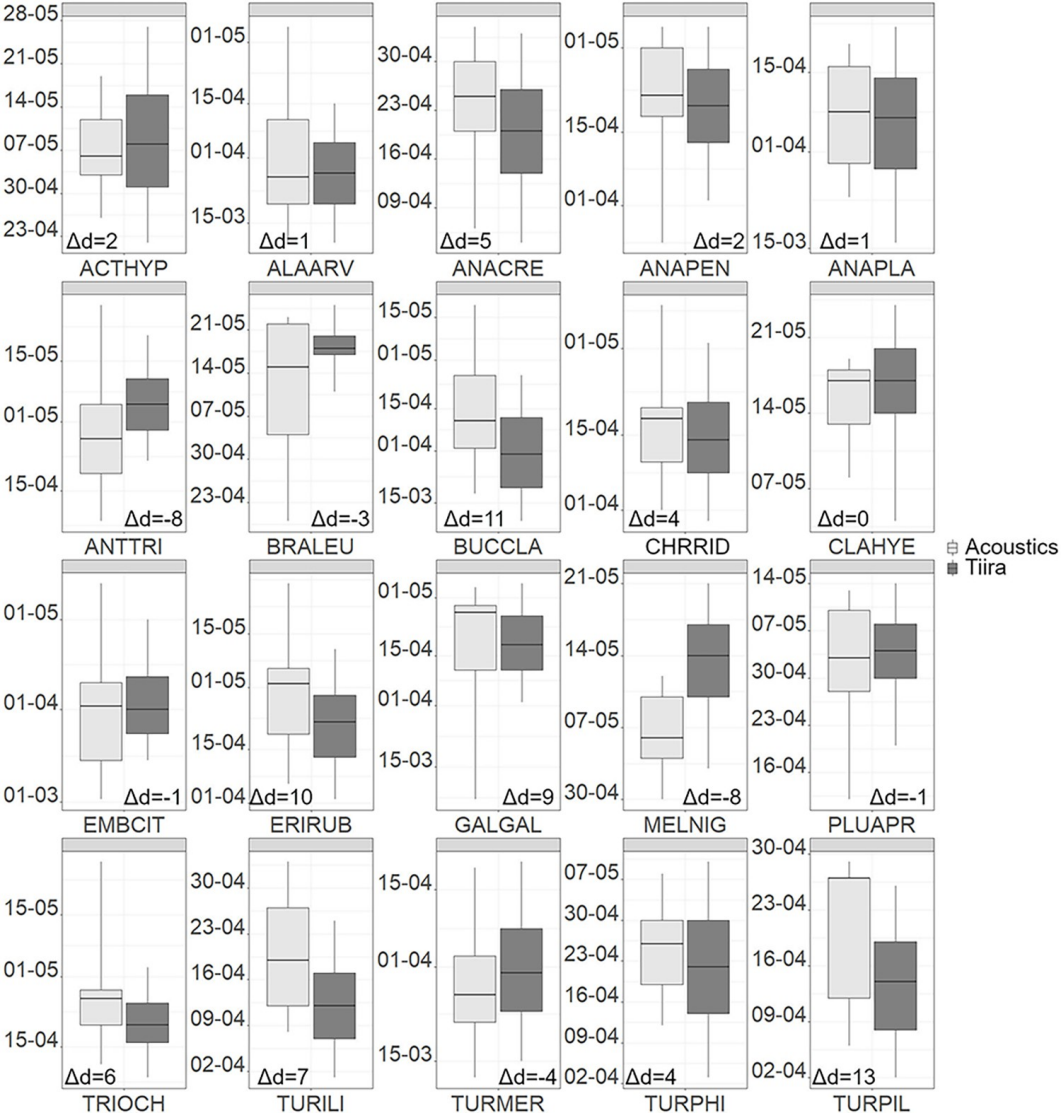
Comparison of medians estimated from acoustic and citizen science data for spring. Differences of medians between acoustic (light grey) and citizen science (dark grey) data are indicated as number of days (Δd). Negative differences mean the citizen science median is later than the acoustic median and vice versa for positive differences. For abbreviation keys see S1 Table.

**Fig 3:**
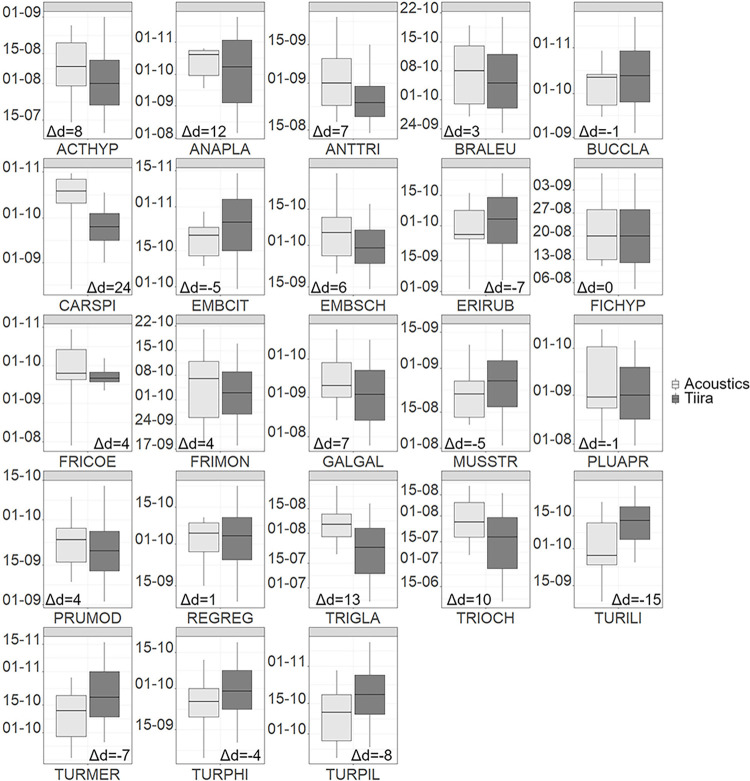
Comparison of medians estimated from citizen science and acoustic data for autumn. Differences of medians between acoustic (light grey) and citizen science (dark grey) data are indicated as number of days (Δd). Negative differences mean the citizen science median is later than the acoustic median and vice versa for positive differences. For abbreviation keys see S1 Table.

CS and acoustic medians differed between |0 and 24| with a mean difference of 2.17 days, i.e. the acoustic medians were on average later than the CS medians (Figs 2 and 3, S3 Table).

A difference in medians of |0 or 1| day was found in four of 20 species (25%) in spring and 4 of 23 species (17%) in autumn. A difference of |2–5| days was found in eight species (40%) in spring and in seven species (30%) in autumn. Eight species differed by |6–15| days in spring (40%) and 11 in autumn (48%). The largest difference was exhibited by the Eurasian siskin in autumn, whose acoustic median was 24 days later than the CS median. Other large differences were found in the Goldeneye (*Bucephala clangula*) (11) and Fieldfare (13) in spring, and in the Mallard (12), Wood sandpiper (13) and Redwing (-15) in autumn.

**Fig 4:**
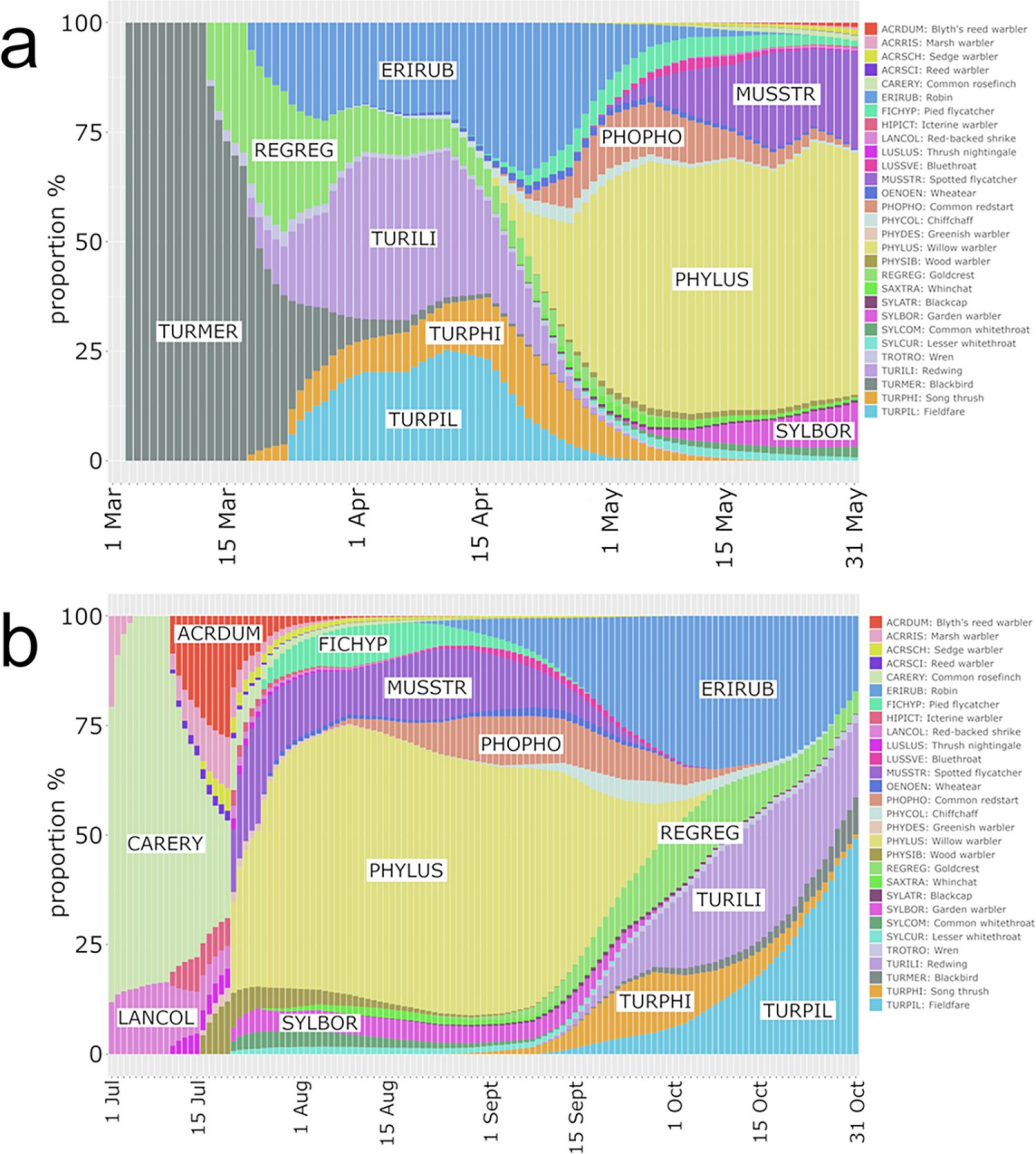
Proportion of nocturnal passerine migrants during spring and autumn season. Proportions of 48 passerine species during spring (a) and autumn (b) migration according to their population sizes and citizen science migration schedules. The most abundant species are labelled, and abbreviation keys are given on the right.

Finally, the proportions of the different species with respect to their population size and the overall passerine, wader and waterfowl populations as determined by bird censuses and CS migration schedules are shown in Fig 4 (passerines) and S2 Fig (waders and waterfowl).

### Radar observations vs. acoustic data

#### Spring

**Fig 5:**
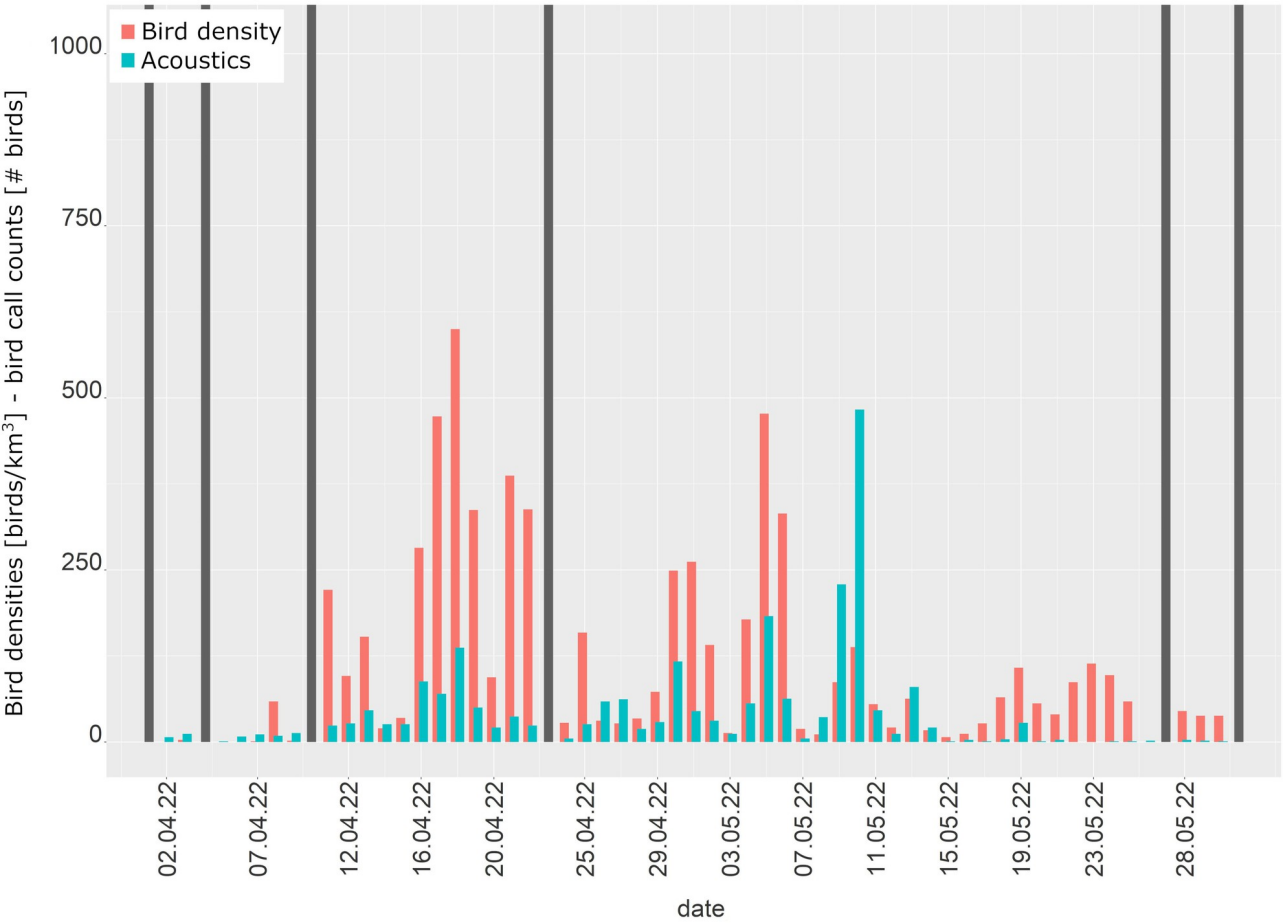
Bird densities from the weather radar and number of calling bird individuals. Bird densities from the Vihti weather radar (red) and the total daily number of calling bird individuals (green) from 1 April to 31 May 2022. Grey bars indicate data unavailability.

Six nights were excluded in spring and four nights in autumn because of technical problems or precipitation either in radar or acoustic data. Acoustic calls increased early April and ceased almost completely in the first half of May, while migration intensities measured by the weather radar started to increment from about mid-April and continued until end of May (Fig 5).

There was a rather weak, but significant correlation between the sum of calling individuals of all bird species and bird densities for the entire spring season (see Table 1).

**Table 1:** Correlation statistics for the comparison of the radar bird densities and the acoustic counts in the spring season 2022.

Species groups and height layers	season	Spearman’s ρ	F	R^2^	p-value
**all species & all height layers**	**All spring**	0.54	7.98	0.13	0.01
	**April**	0.72	23.99	0.50	<0.001
	**May**	0.38	4.47	0.14	0.04
**passerines only & all height layers**	**All spring**	0.47	18.85	0.26	<0.001
	**April**	0.79	8.84	0.27	0.01
	**May**	0.41	6.29	0.19	0.02
**all species & lowest layer**	**All spring**	0.73	9.84	0.16	<0.01
	**April**	0.78	27.60	0.55	<0.001
	**May**	0.67	6.14	0.18	0.02
**passerines only & lowest layer**	**All spring**	0.61	44.87	0.46	<0.001
	**April**	0.79	19.57	0.46	<0.001
	**May**	0.54	25.26	0.47	<0.001

Spearman’s rank correlation coefficient, F-statistic, R^2^ and p-value for each combination of all bird species or passerines only in acoustic data and the entire height range or the lowest layer only in radar bird densities in spring (n = 55 nights), April (n = 26 nights) and May (n = 29 nights).

For the early spring period from 1–30 April correlation was strong for all species and height layers, but weak later from 1–31 May (Table 1). In the passerine correlation analyses, the entire spring season showed a weaker correlation with weather radar data compared to all species (Table 1). For the early spring period from 1–30 April correlation was stronger than for all species and from 1–31 May it was clearly weaker than April for passerines and slightly stronger than May for all species.

The bird densities from the lowest height layers showed a much stronger correlation with all acoustic species than the entire scan range of four kilometres for the entire spring season as well as for April and May separately (Table 1). However, when reducing the acoustic species range to passerines for the lowest level, correlation of overall spring and May dropped. Only the April correlation remained almost equal.

In summary, strongest correlations were obtained using the lowest layer only, either for the entire species range or for passerines only.

Flight altitudes in spring extended up to nearly 2000 m, with the highest concentration up to about 600 m and a median flight altitude (50^th^ percentile) in the layer of 200–400 m in both April and May (S3A Fig).

Passerine species with more than 100 calls and at least 10 call nights in the spring season were Redwing and Song thrush. The Redwing (Spearman’s rho = 0.74, F = 10.18, R^2^ = 0.35, p-value <0.01, n = 25) showed a stronger correlation and better fit with the radar data than the Song thrush (Spearman’s rho = 0.57, F = 4.47, R^2^ = 0.12, p-value = 0.04, n = 38).

#### Autumn

**Fig 6:**
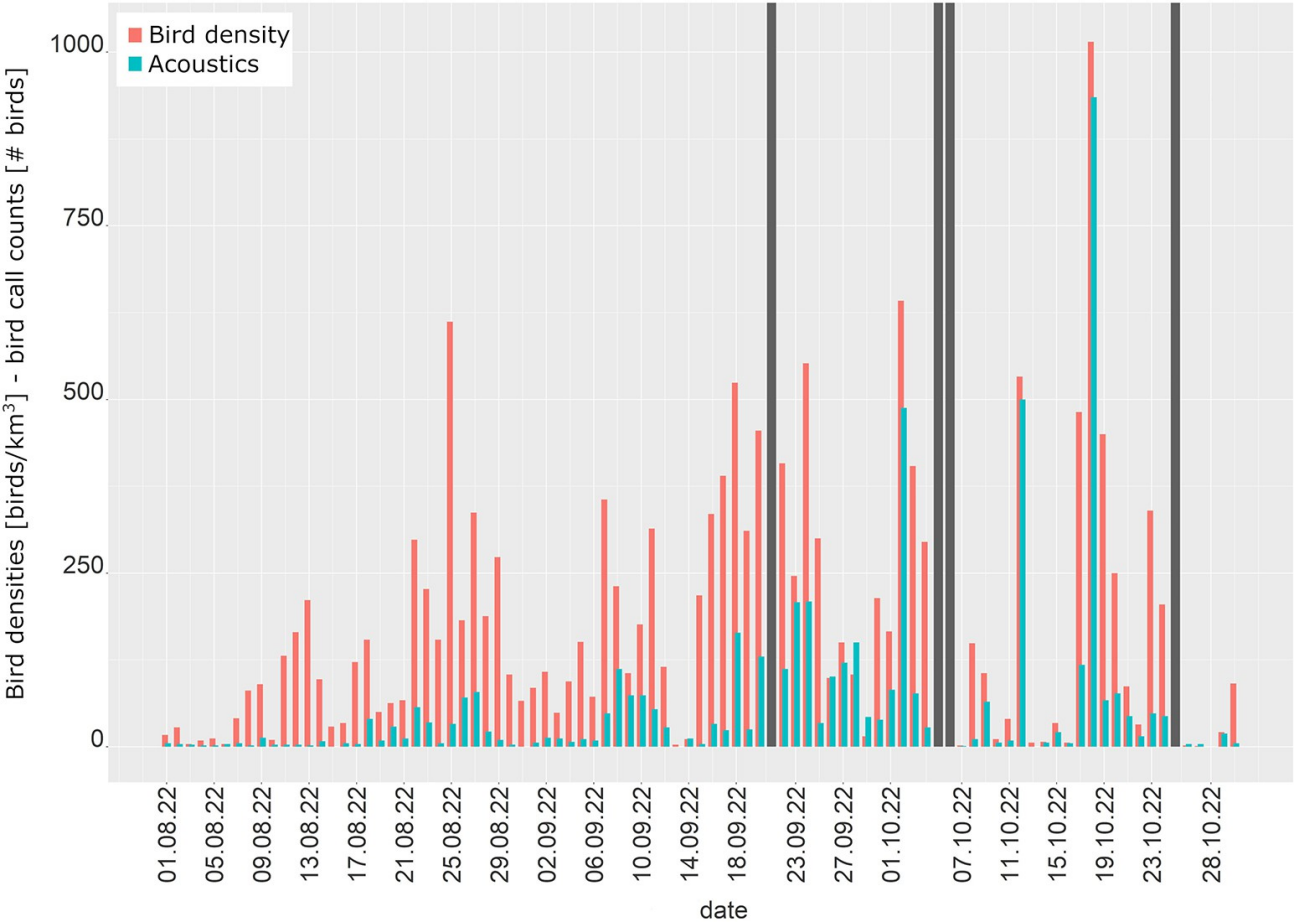
Bird densities from the weather radar and number of calling bird individuals. Bird densities from the Vihti weather radar (red) and the total daily number of calling bird individuals (green) from 1 August to 31 October 2022. Grey bars indicate data unavailability.

Acoustic and radar-derived migration intensities started to increase in the first half of August and especially in acoustic records there was an increment towards later autumn season (Fig 6).

There was a significant correlation between the sum of all calling species and bird densities for the entire autumn season, for the main season of long-distance migrants from 1 Aug to 9 Sept and for the remaining season between 10 Sept to 30 Oct (Table 2).

**Table 2:** Correlation statistics for the comparison of the radar bird densities and the acoustic counts in the autumn season 2022.

Species and height layers	season	Spearman’s rho	F	R^2^	p-value
**all species & all height layers**	**All autumn**	0.72	102.05	0.55	<0.001
	**1 Aug– 9 Sept**	0.59	14.33	0.27	<0.001
	**10 Sept– 31 Oct**	0.78	68.73	0.60	<0.001
**passerines only & all height layers**	**All autumn**	0.75	48.21	0.36	<0.001
	**1 Aug– 9 Sept**	0.69	13.59	0.26	<0.001
	**10 Sept– 31 Oct**	0.78	24.12	0.35	<0.001
**all species & lowest layer**	**All autumn**	0.69	56.25	0.40	<0.001
	**1 Aug– 9 Sept**	0.6	11.11	0.23	<0.01
	**10 Sept– 31 Oct**	0.79	57.43	0.56	<0.001
**passerines only & lowest layer**	**All autumn**	0.73	31.93	0.27	<0.001
	**1 Aug– 9 Sept**	0.72	11.21	0.23	<0.01
	**10 Sept– 31 Oct**	0.8	22.18	0.33	<0.001

Spearman’s rank correlation coefficient, F-statistic, R^2^ and p-value for each combination of all bird species or passerines only in acoustic data and the entire height range or the lowest height layer (0–200 m) only in radar bird densities in autumn (n = 87 nights), 1 Aug–9 Sept (n = 40 nights) and 10 Sept–31 Oct (n = 47 nights).

In the passerine correlation analyses, the entire autumn season exhibited a slightly stronger and significant correlation with weather radar data compared to all species considering all heights (Table 2). For the early autumn period from 1 Aug to 9 Sept correlation was clearly stronger than for all species and between 10 Sept to 30 Oct the correlation coefficient was the same as for all species together in the entire height range (Table 2).

The bird densities from the lowest height layer showed a weaker correlation with all species of the acoustic data than the entire height range of four kilometres for the entire autumn season, and a slightly improved correlation for August-early September and for late September-October (Table 2) separately. Reducing the acoustic species range to passerines for the lowest level improved the correlation of both early and late autumn seasons, though not for overall autumn.

Contrary to spring, the strongest correlations were thus obtained for the passerine species composition, either for the entire height range (all autumn) or using the lowest layer only.

Flight altitudes in autumn were more widely distributed than in spring, i.e. up to about 2500 m, with a median flight altitude in the layer of 400–600 m (S3B Fig).

The following species with at least 100 call records and 10 call nights were included in the species-specific passerine analyses: Tree pipit, European robin, Redwing, Common blackbird and Song thrush. Blackbird and Redwing showed the strongest correlation and the best fit (Table 3), while Tree pipit, European robin and Song thrush exhibited a moderate correlation with radar data.

**Table 3:** Correlation statistics for the comparison of the radar bird densities and the five most abundant calling passerines in the autumn season 2022.

Species	Spearman’s rho	F-statistic	R^2^	p-value	n (number of nights)
Tree pipit (*A*. *trivialis*)	0.58	9.95	0.24	<0.01	33
European robin (*E*. *rubecula*)	0.54	6.08	0.12	0.01	46
Redwing (*T*. *iliacus*)	0.76	17.85	0.38	<0.001	31
Blackbird (*T*. *merula*)	0.72	20.33	0.40	<0.001	32
Song thrush (*T*. *philomelos*)	0.53	8.23	0.19	0.01	37

Spearman’s rank correlation coefficient, F-statistic, R^2^ and p-value for the most abundant passerine species in acoustic data and the lowest layer in radar bird densities in autumn.

## Discussion

### Citizen science vs. acoustic data

Acoustic data showed high variability in qualitative and quantitative content. While some few species agreed relatively well with CS migration schedules in spring and autumn, most species accorded only in one season with CS data or exhibited an indistinct occurrence pattern without any particular peaks which would be indicative of migration.

The observed call patterns may be shaped by several factors. Species abundance certainly has an impact on records. In some cases, low numbers of records are most likely related to the actual low abundance of some species, such as the Little grebe (*Tachybaptus ruficollis*), Gadwall (*Anas strepera*) or Ortolan bunting (*Emberiza hortulana*). The probability that a rare species flies over the narrow sampling area of a microphone is minute. The low numbers of Ortolan buntings (four individuals), but also Lapland (22 individuals) and Snow buntings (26 individuals), can be compared to records in [14] who observed several hundreds or even thousands of calling individuals of these species in single events in the same Helsinki area at night. Also, [12] reported that “one of the most common calls” at night pertained to the Ortolan bunting. So, the practical absence of these species is a true reflection of their actual population decline which has been observed in recent decades, in Ortolan bunting even 99% in 40 years [42, 43]. However, many abundant species were either completely missing from the dataset or were present in low numbers. The Redstart (two calling individuals) and Pied flycatcher (23 calling individuals) were clearly underrepresented considering their estimated populations of about half a million breeding pairs each only in Finland [24]. There were no records of *Sylvia spp*., *Acrocephalus spp*. and *Phylloscopus spp*. which is in line with observations by [14]. For instance, the Willow warbler *(Phylloscopus trochilus)*, with an estimated Finnish breeding population of about 7–11 million breeding pairs, represents about 50% of nocturnally migrating passerine birds in early September, but acoustic data did not contain it. The ten most abundant *Sylvia*, *Acrocephalus* and *Phylloscopus* species (i.e. *Sylvia atricapilla/borin/communis/curruca; Acrocephalus schoenobaenus/scirpaceus; Phylloscopus collybita/sibilatrix/trochiloides/trochilus)* together constitute about 47% of the nocturnally migrating passerine populations and 68% of the nocturnally migrating long-distance migrants in the southern Finnish airspace (Weisshaupt and Koistinen [Unpublished]). This disagreement in numbers of calls and numbers of birds has also been found in previous studies in the same area [14] or elsewhere in Europe or the US (e.g. [8] and citations therein, [44]). Contrary to the statement by [8] that “patterns of call counts across seasons and years are often consistent”, we found that patterns are often inconsistent or variable across seasons and between species. Several species were recorded either in spring or autumn, such as the Tree pipit, Dunnock or several duck species. The seasonal emphasis of duck calls on spring is due to the male calls predominantly uttered in spring. Some species were only recorded in single or some years, e.g. Eurasian teal (together with the Mallard the most common duck species in Finland) and Eurasian wigeon called only several times in one year in autumn; the majority of crane calls from spring and autumn stem from the same year. Even though there is evidence for changing call rates during the night [44, 45], the reason for this seasonally (and annually) diverging call pattern remains indeterminate. Perhaps such findings remained undetected in previous acoustic studies because of low species numbers, few sampled seasons and years (e.g. [9]) or because species-level data was not assessed [45]. To some extent seasonal differences may be explained by different migration routes in spring and autumn influenced additionally by weather, e.g. many arctic waterfowl species take a more southern route over the Baltic Sea or Estonia in autumn than in spring [46]. Arctic migration can, however, cross Finland in easterly winds. It cannot explain, though, the seasonal differences for all species concerned. The use of multi-site data may help understand and exclude spatial biases in the outcomes. Migration timing (day or night) certainly also played into the presence or absence of species, e.g. day migrants such as finches and wagtails were clearly underrepresented in the data considering their populations. Finally, the microphone and its detection range together with meteorological conditions have an impact on the numbers of birds observed. In Europe, nocturnal passerine migration can be distributed vertically across several kilometers, with highest concentrations between about 400–1000 m [47]. The exact detection range of sound recording devices is often unknown [48]. Based on comparisons of human hearing and audio recordings by one of the authors during day migrations, in the present case the detection range of the microphone can be assumed to be at least equivalent to human hearing capacity or at times superior (besides differences in human hearing there might be a bias through variable listening endurance and risk of distraction in human observers, which does not affect the recording device). The detection range can be cautiously assumed to be at least several tens to hundreds of meters depending on the atmospheric attenuation of the sound, ambient noise (e.g. from rain or wind), the frequency of the bird call, the direction in which the call is uttered and so on [45, 49]. Additionally, birds’ flight distance to the stationary recorder varies with weather conditions, e.g. wind direction and speed, fog or rain might force birds to alight or fly at lower altitudes (e.g. [50]). Consequently, they enter (or not) the range of the microphone, and their calls would be captured more easily. In the present study, we did not investigate the impact of weather variables on call rates. Seasonal differences in flight altitudes are a known phenomenon (e.g. [47, 51]) and have also been observed in the Baltic Sea [52]. Higher flight altitudes in spring as found in these studies could explain the silence in spring because birds would evade being recorded. However, in the present work, radar data from 2022 showed that birds were flying on average at slightly higher altitudes and vertically more widely distributed in autumn compared to spring. So, flight altitudes do not appear to explain the discrepancies. Interestingly, the Redwing did not correlate as well with CS schedules as the Song thrush in autumn even though it had 15% more call records than the Song thrush. The migration peaks were slightly shifted, which weakened correlations. The reason behind this divergence remains unknown as both species are highly vocal and the Redwing call should be equally well detected as the Song thrush call by both recording devices and citizen scientists.

So, there is a variety of unquantified factors that have an impact on data quality and quantity, and it can be difficult to separate true negative observations from false negatives without previous knowledge of local migrant composition through complementary data sources. That is, positive acoustic detection of a species is a valid piece of live presence-only data and often the only information on species level at night. For instance, it may deliver valid information on spatial migration distribution of eligible species as in [53]. Presence-absence or quantitative inferences require, however, critical evaluation of the acoustic data for each species for the reasons discussed above.

Qualitative yield of information was significantly improved by accruing data over several years. In the present study, data of one year often did not suffice to depict migration phenology of species. In contrast, multiple years of sound recordings can already deliver valuable data on timing of migration in spring and/or autumn for some species, e.g. some waders like Common sandpiper, some waterbird species, and most thrush species. Multi-year data may even deliver quantitative phenologies, i.e. the average peak migration period in one or both seasons. Median analyses showed relatively good agreement between CS and acoustic outcomes. Medians of about half of the species in both spring and autumn differed by 5 days or less. Large differences of more than 10 days may occur for several reasons. There may be sedentary or resting populations which may obscure true migration movements, e.g. in the Mallard. Also, the calling activity may vary during the season and according to weather [8, 13], which may bias the counts. Small numbers of sample nights together with some extreme acoustic events may shift the 50^th^ percentiles. Extreme migration events may have a large impact on the percentiles. For instance, the 50^th^ percentile of the Fieldfare in spring coincides with its 75^th^ percentile because of an exceptional event on 27 April 2020 with 87 individuals, while nightly counts were otherwise below 20 individuals and most often zero. Such events carry more weight in low count numbers compared to the often immense bird numbers in CS data. Large differences may also result from “level" acoustic phenologies through small numbers of calling individuals per night despite 10 call nights or more as e.g. in the Wood sandpiper. The largest difference of 24 days was found in the Eurasian siskin. The challenge with Siskins is their irregular and irruptive migration with high annual variation together with the fact that it is a day migrant. Acoustics and day observations may not always capture active migration and annual variability in migration timing can be huge. A trickier case is the Redwing and its 15 days of discrepancy from the CS median in autumn. One would anticipate the Redwing to perform like the Song thrush, which is also a highly vocal nocturnal migrant of about the same size, so the detectability and observability by day and night should be the same. However, the median of the Song thrush differed by only 4 days from the CS median. The reason for the large difference in the Redwing remains unknown.

Even though migration phenologies of several species were comparable to CS phenologies, the range of eligible species is rather small and boils down to about 25 of 81 in the present study. Comparing the informational yield of acoustics with visual CS observations, CS observations cover a far broader range of species of both diurnal and nocturnal migrants than acoustics. Acoustics do not alleviate the lack of data for several clandestine migrants, such as Blyth’s reed warbler (*A*. *dumetorum*) and Marsh warbler (*A*. *palustris*), which simply disappear unseen and unheard at some point in late summer. In such cases only long-term ringing data with comparable daily capture effort can provide seasonal distributions [21].

### Radar vs. acoustic data

#### Spring

Radar data showed a moderate correlation with acoustic data for the entire spring season. The separation of early (April) and late spring (May) revealed a stronger significant correlation in April, when mainly short-distance passerines, amongst other highly vocal thrush species, migrate, and a weak correlation in May. In general, there were only few acoustic species records, with only four species exhibiting more than 100 individuals during the spring period, the Barnacle goose, Common scoter and the passerine species Redwing and Song thrush. Low-altitude spring migration measured by the radar correlated better with acoustics than the entire altitude range for both passerines and the entire species range. In April, low-altitude migration of passerines showed strongest correlation with radar data, which is most likely due to the presence of the Redwing and Song thrush. Of these two species, the Redwing showed a stronger correlation with radar data than the Song thrush. The reason for that remains unknown given the similarity of the two species in acoustic and physical observability, as well as migration strategy (nocturnal) and timing. The umbrella effect by the thrushes as a proxy for overall migration intensity is then diluted in May with the massive arrival of an increasing number of silent migrants, especially passerines, but also waterfowl. In May, the entire species range performed better in correlation analyses compared to passerines, which indicates a prominent role of arctic waterfowl (and waders) that are moving then in great numbers through the area of Helsinki and the Gulf of Finland. Overflying waterfowl flocks are heard at higher altitudes than passerines (authors [Unpublished]) and they are well visible to the radar [6]. Therefore, they seem a reasonable factor to explain the correlation results given the very few vocal passerines in May and the weaker passerine correlations at low altitude.

#### Autumn

The correlations between the radar and acoustic data were significant, though weaker in August and beginning of September compared to the later autumn period and the entire autumn season. The strongest correlations were found for passerines, either in the entire height range (entire autumn) or at the lowest level (partial autumn seasons), with the strongest correlation in late autumn. These results suggest that passerines play a dominant role in radar echoes, with negligible impact from non-passerine migration, if any. Summer and early autumn are the peak migration season for many wader species, whose calls were regularly caught in low numbers by the microphone. However, calling waders represented only a fraction of the total number of calling individuals, i.e. too few and too random to reflect overall migration observed by radar. Additionally, passerine populations outnumber waders by far and waders are often masked by the migrating passerines in the radar data at night. Interestingly, a rather strong correlation was achieved in early autumn, even though most of the nocturnal long-distance migrants remained unappreciated by acoustics. This suggests that the total of passerine species recorded in that period, such as Spotted flycatcher and Tree pipit,—despite moderate species-specific correlation for the latter—sufficed as proxies for other migrants travelling under similar conditions.

In the later period of September and October, when several highly vocal species were on the move, correlations between acoustic counts and bird densities were strongest. When looking at the most numerous calling passerine species, i.e. the best candidates for a good fit with the radar bird densities, the Redwing and the Blackbird performed best. Unexpectedly, the abundant Song thrush did not correlate well with radar data, even though its migration period starts after the main period of long-distance migrants, and it had most calling individuals and call nights after the Redwing. Despite the rather moderate number of calling individuals, the correlation of the Blackbird was surprisingly strong, but this good result was probably due to the increasing absence of silent species in late autumn. The Tree pipit with about three times as many calling individuals as the Blackbird, but with a much weaker correlation, migrates in late August and September when many silent species are still dominating the nocturnal migration. So, the number of calling individuals and call nights do not necessarily warrant good correlations with radar data, which was also observed by [45]. The pivotal role of passerines also later in autumn fits well with the fact that arctic migrants typically take a more southern route in autumn compared to spring avoiding mostly passage through the Helsinki area. Only in strong easterly winds does arctic waterfowl, especially Barnacle and other goose species, pass over southern Finland.

## Conclusions

Acoustic data can deliver valuable nocturnal live taxonomical information for eligible migrants in suitable occasions. However, acoustic data is practically unable to reflect the overall migration flux in an aerial volume simultaneously sampled by radar. Thus, the numerous quantitative and qualitative uncertainties attached to sound recordings make it a challenging data source in migration research and derived applications. In particular, absence of calls does not necessarily equate to true species absence and thus bird flux, especially if suitable umbrella species are absent. It is essential to identify an appropriate niche for acoustic data to effectively integrate it with added value into migration studies. Most likely the highly vocal and abundant species, particularly Song thrush and Redwing, offer room for innovative complementary applications in radar and generally migration research, if they are dominating a migration event. We expect the present findings to be valid also for other parts of Europe given the distribution range of the vocal and silent species recorded in this study.

## Supporting information

S1 Fig Migration phenologies estimated from citizen science and acoustic observations. Nightly mean numbers of calling individuals and standard deviations for the years 2019–2022 (blue bars) and citizen science migration phenologies (black line) from the bird portal *Tiira* in pentads.
(PDF)

S2 Fig Proportions of wader and waterfowl population sizes during spring and autumn migration. Proportions of 21 wader and 17 waterfowl species of populations during spring and autumn migration according to their population sizes and citizen science migration schedules. The most abundant species are labelled.
(PDF)

S3 Fig Flight height distributions of migrating birds as observed by weather radar during spring and autumn 2022. Mean proportions (and standard deviations) of birds (%) in 200-m height layers in the weather radar in Vihti, Finland, in spring (a) and autumn (b) 2022.
(TIF)

S1 Table Species list from citizen science and acoustic data, respective migrant population sizes and number of calling bird individuals used in the present study. Spring population size: estimated migrant population sizes in Southern Finland in spring; autumn population size: estimated migrant population sizes in Southern Finland in autumn; CS data: availability of citizen science migration schedules; Number of calling individuals in acoustic data: number of calling individuals in acoustic data from 2019–2022 for spring, autumn and in total.
(PDF)

S2 Table Statistical outcomes from the Spearman’s rank correlation and regression analyses for citizen science and acoustic data in spring and autumn season. Acoustic data consist of the four-year dataset from 2019–2022 and species with at least 10 call nights per season were included. For species which did not fulfil this criterion fields are marked as “NA”.
(PDF)

S3 Table 5^th^, 50^th^ and 95^th^ percentiles estimated for spring and autumn season based on the four-year acoustic dataset. Species with at least 10 call nights per season were included. For species which did not fulfil this criterion percentiles are labelled as “NA”.
(PDF)
