# Supplementary material for: Evaluating the potential of bioacoustics in avian migration research by citizen science and weather radar observations
Source: PLoS One. 2024 Mar 8;19(3):e0299463. doi: 10.1371/journal.pone.0299463 (PMC10923479; doi:10.1371/journal.pone.0299463)
Supplement: S1 Fig — Nightly mean numbers of calling individuals and standard deviations for the years 2019–2022 (blue bars) and citizen science migration phenologies (black line) from the bird portal Tiira in pentads. (PDF) [file pone.0299463.s001.pdf]

Supporting Information S1 Fig

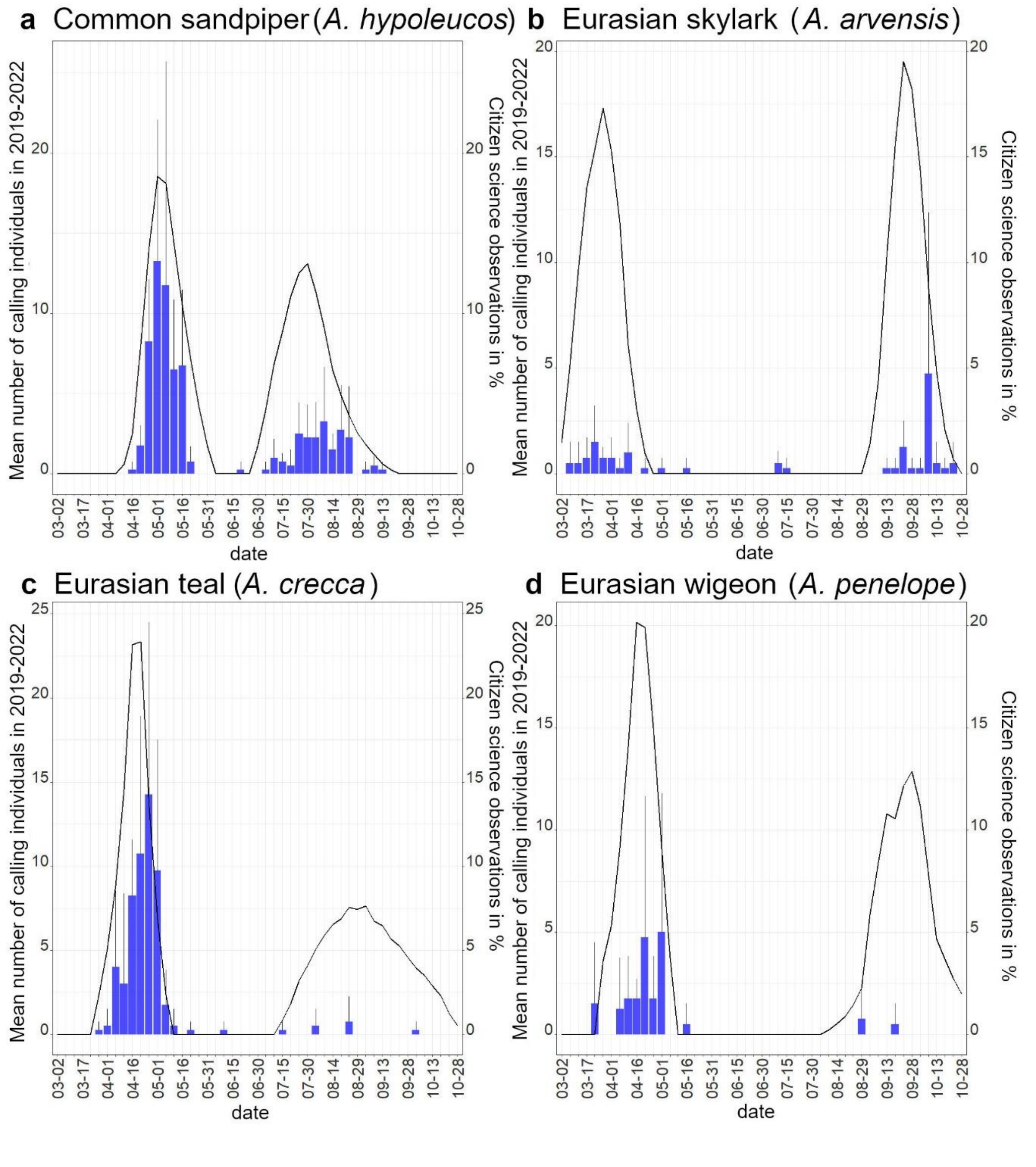

S1a-d Figs. **Migration phenologies estimated from citizen science and acoustic observations.** Nightly mean numbers of calling individuals and standard deviations for the years 2019-2022 (blue bars) and citizen science migration phenologies (black line) from the bird portal *Tiira* in pentads, here for the Common sandpiper (*A. hypoleucos*), Eurasian skylark (*A. arvensis*), Eurasian teal (*A. crecca*) and Eurasian wigeon (*A. penelope*).

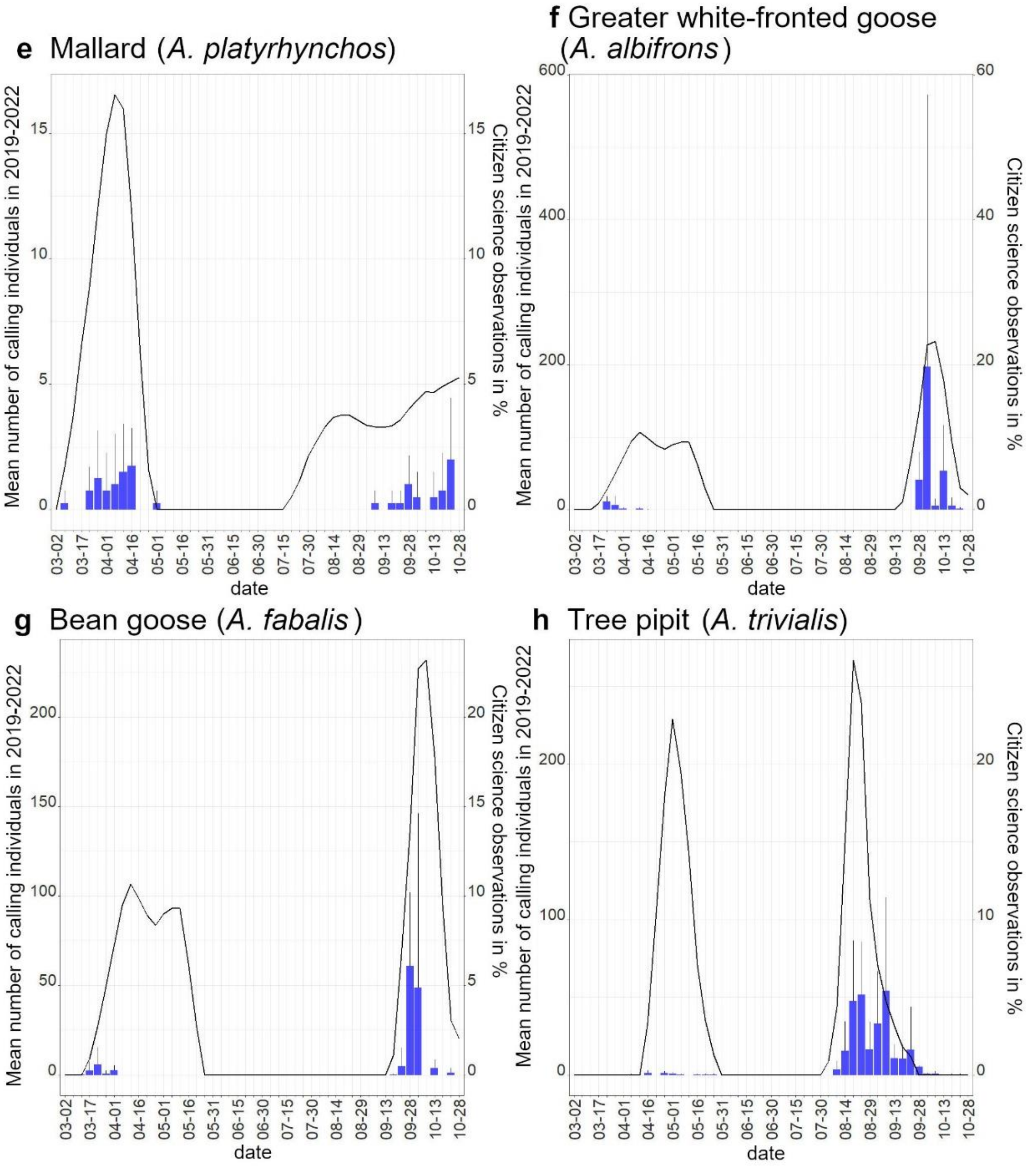

S1e-h Figs. **Migration phenologies estimated from citizen science and acoustic observations.** Nightly mean numbers of calling individuals and standard deviations for the years 2019-2022 (blue bars) and citizen science migration phenologies (black line) from the bird portal *Tiira* in pentads, here for the Mallard (*A. platyrhynchos*), Greater white-fronted goose (*A. albifrons*), Bean goose (*A. fabalis*) and Tree pipit (*A. trivialis*).

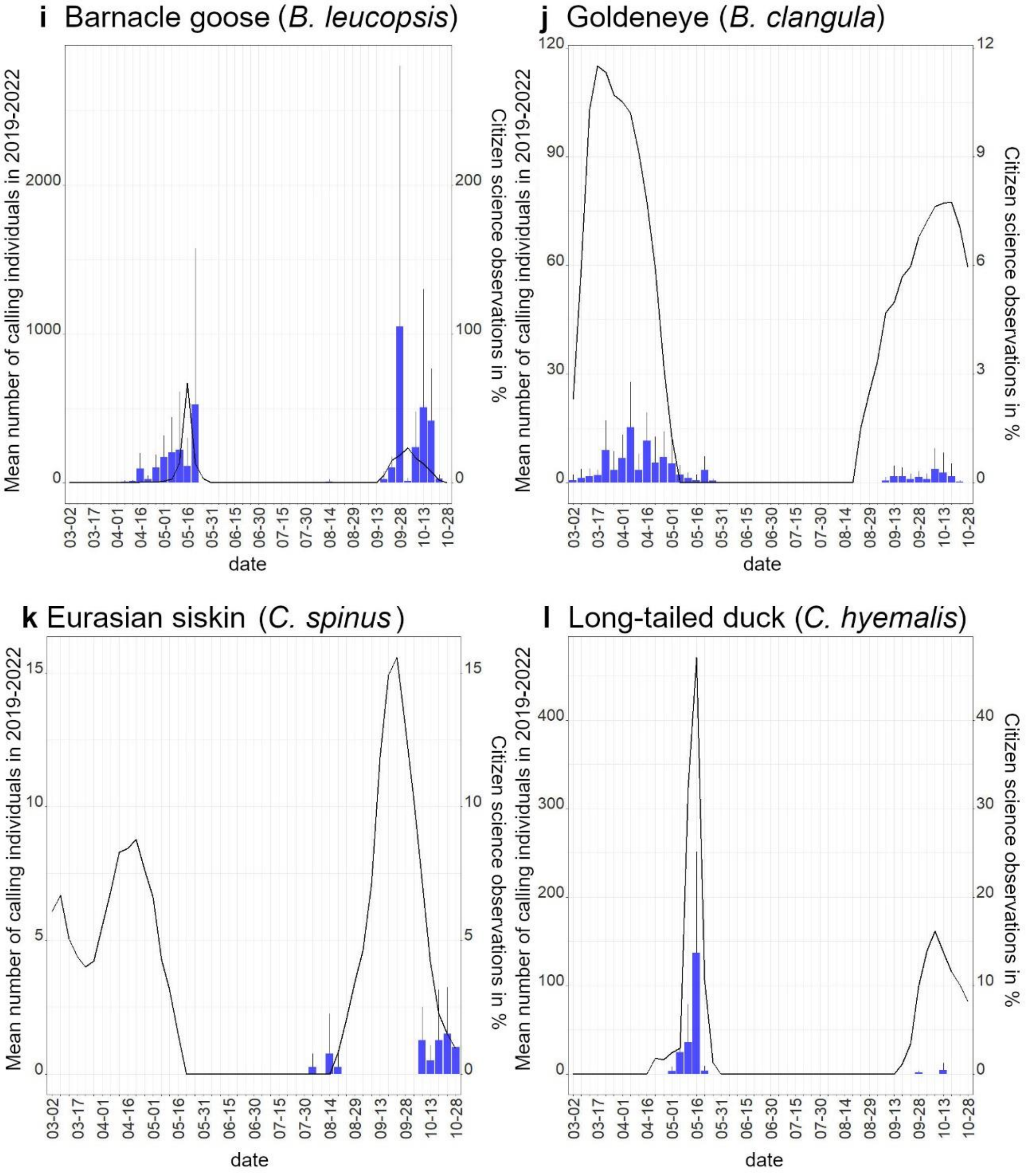

S1i-l Figs. **Migration phenologies estimated from citizen science and acoustic observations.** Nightly mean numbers of calling individuals and standard deviations for the years 2019-2022 (blue bars) and citizen science migration phenologies (black line) from the bird portal *Tiira* in pentads, here for the Barnacle goose (*B. leucopsis*), Goldeneye (*B. clangula*), Eurasian siskin (*C. spinus*) and Long-tailed duck (*C. hyemalis*).

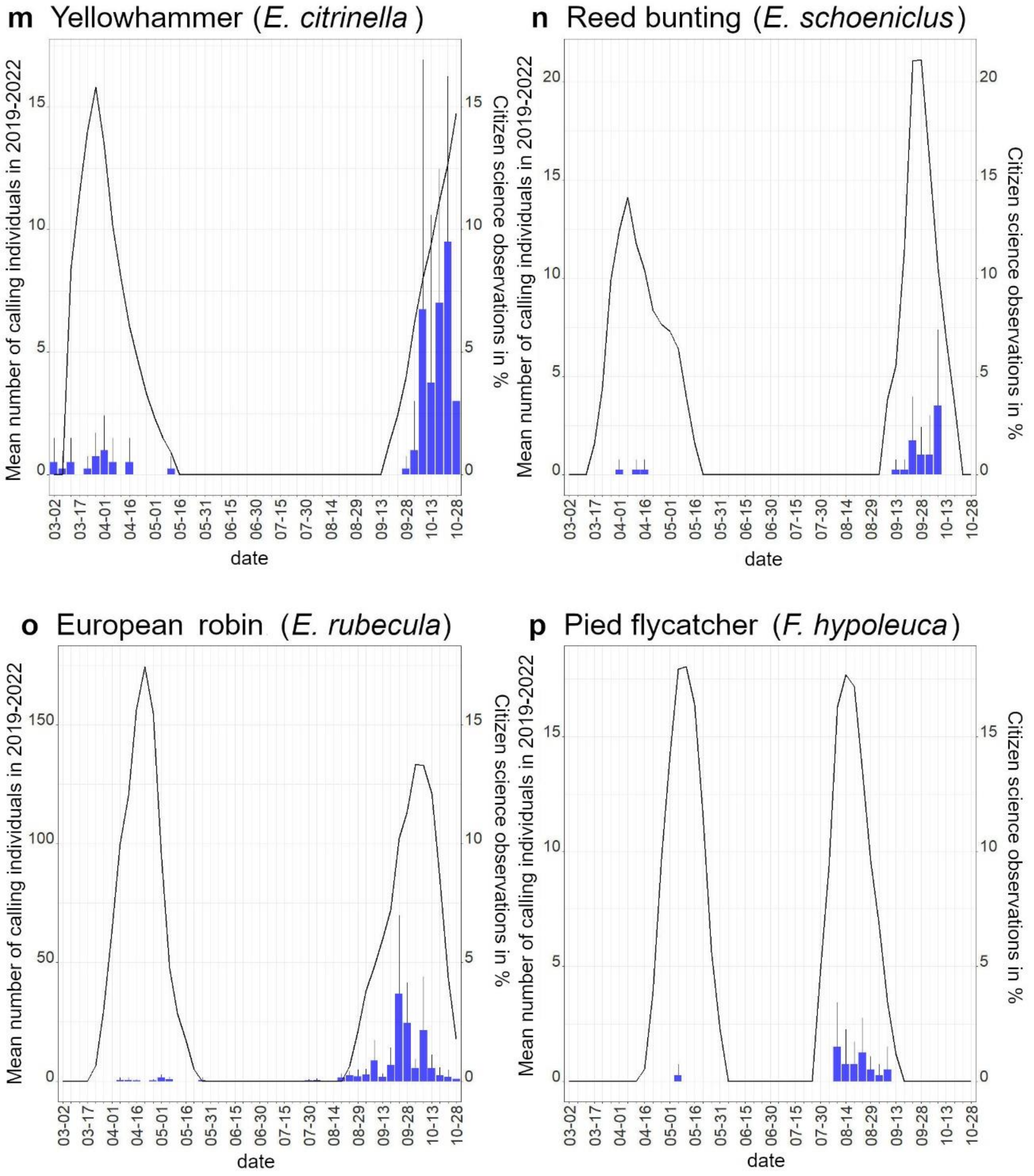

S1m-p Figs. **Migration phenologies estimated from citizen science and acoustic observations.** Nightly mean numbers of calling individuals and standard deviations for the years 2019-2022 (blue bars) and citizen science migration phenologies (black line) from the bird portal *Tiira* in pentads, here for the Yellowhammer (*E. citrinella*), Reed bunting (*E. schoeniclus*), European robin (*E. rubecula*) and Pied flycatcher (*F. hypoleuca*).

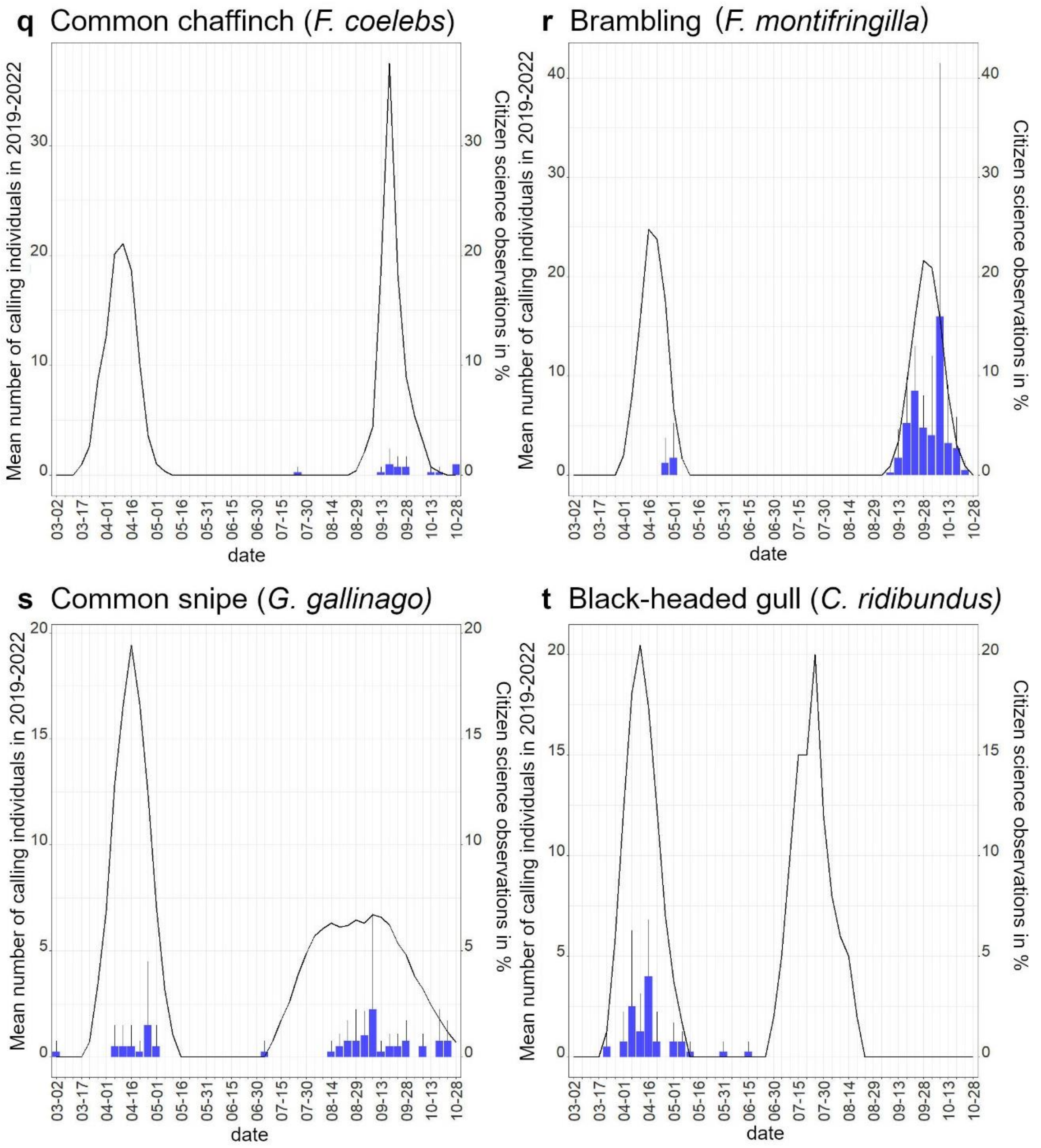

S1q-t Figs. **Migration phenologies estimated from citizen science and acoustic observations.** Nightly mean numbers of calling individuals and standard deviations for the years 2019-2022 (blue bars) and citizen science migration phenologies (black line) from the bird portal *Tiira* in pentads, here for the Common chaffinch (*F. coelebs*), Brambling *(F. montifringilla*), Common snipe (*G. gallinago*) and Black-headed gull (*C. ridibundus*).

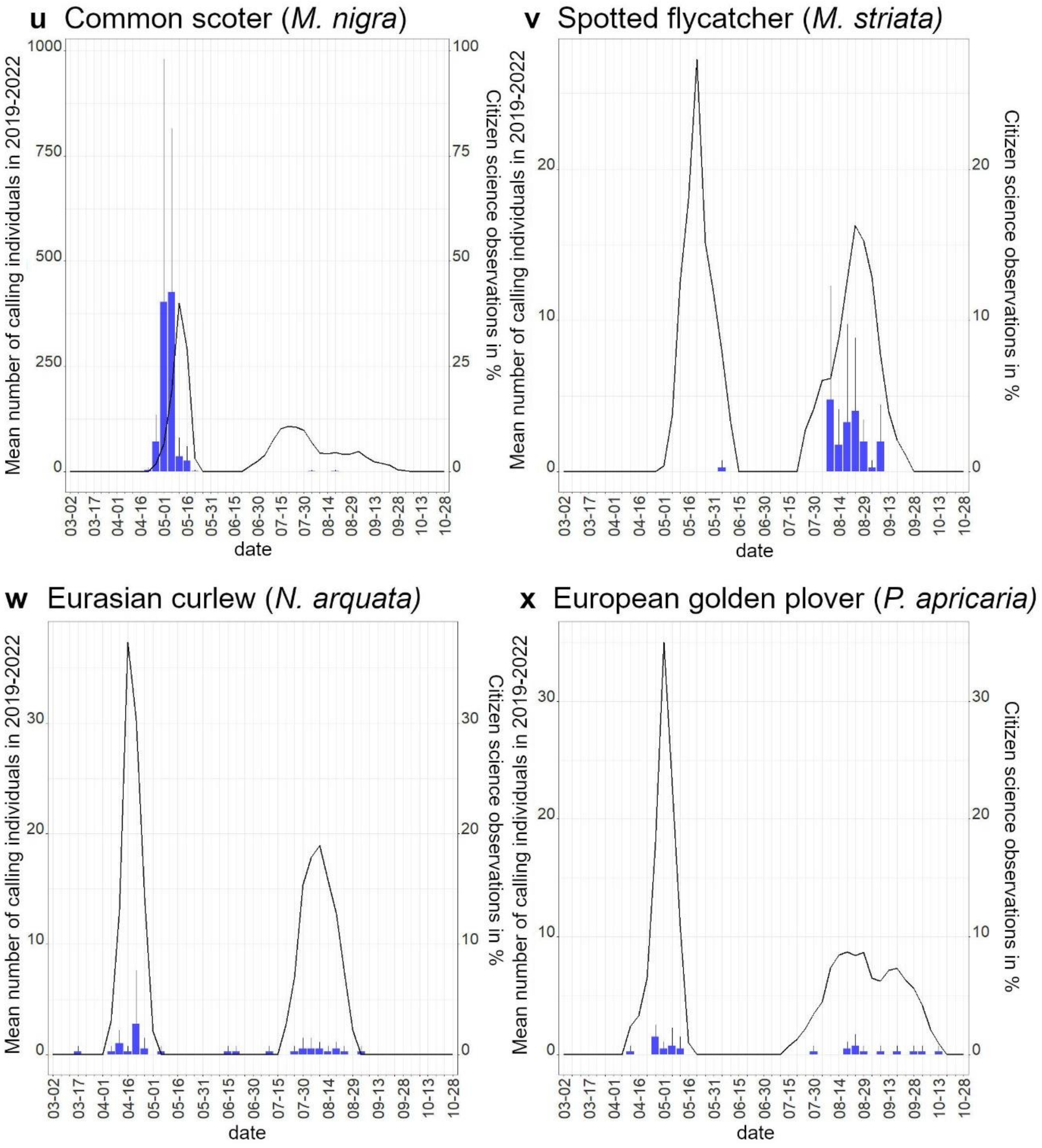

S1u-x Figs. **Migration phenologies estimated from citizen science and acoustic observations.** Nightly mean numbers of calling individuals and standard deviations for the years 2019-2022 (blue bars) and citizen science migration phenologies (black line) from the bird portal *Tiira* in pentads, here for the Common scoter (*M. nigra*), Spotted flycatcher (*M. striata*), Eurasian curlew (*N. arquata*) and European golden plover (*P. apricaria*).

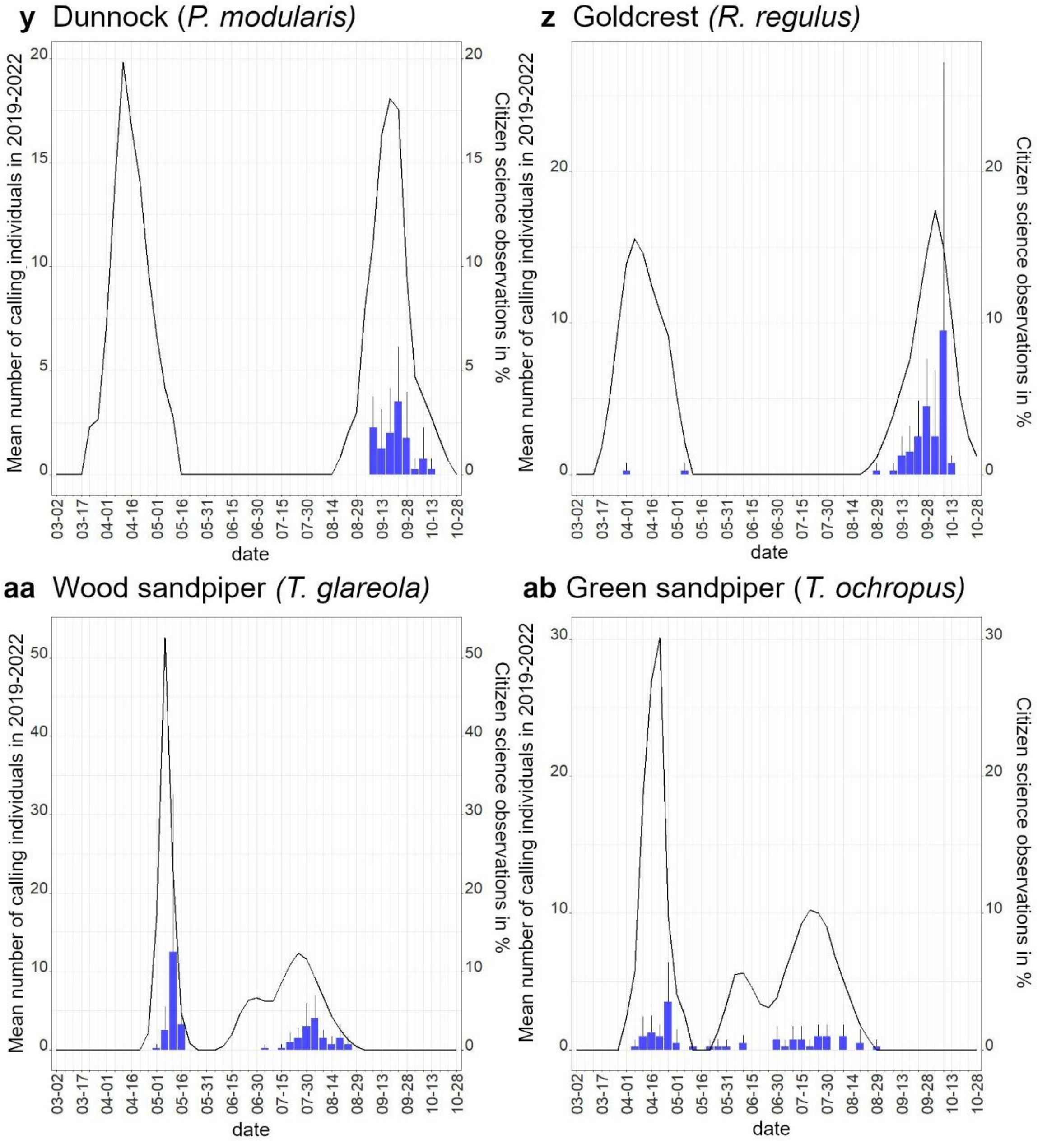

S1y-ab Figs. **Migration phenologies estimated from citizen science and acoustic observations.** Nightly mean numbers of calling individuals and standard deviations for the years 2019-2022 (blue bars) and citizen science migration phenologies (black line) from the bird portal *Tiira* in pentads, here for the Dunnock (*P. modularis*), Goldcrest (*R. regulus*), Wood sandpiper (*T. glareola*) and Green sandpiper (*T. ochropus*).

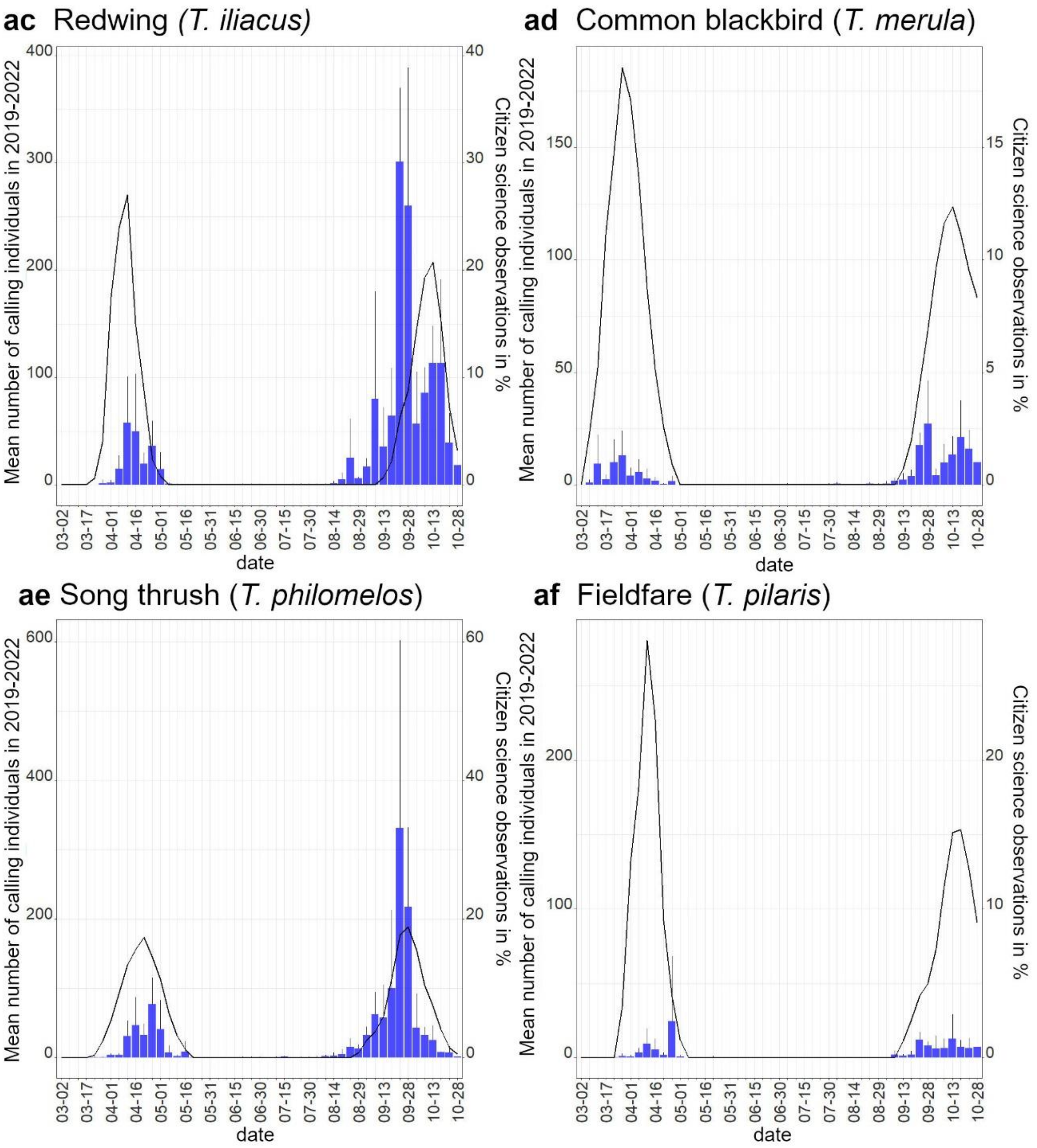

S1ac-af Figs. **Migration phenologies estimated from citizen science and acoustic observations.** Nightly mean numbers of calling individuals and standard deviations for the years 2019-2022 (blue bars) and citizen science migration phenologies (black line) from the bird portal *Tiira* in pentads, here for the Redwing (*T. iliacus*), Common blackbird (*T. merula*), Song thrush (*T. philomelos*) and Fieldfare (*T. pilaris*).
