# Supplementary material for: Evaluating the potential of bioacoustics in avian migration research by citizen science and weather radar observations
Source: PLoS One. 2024 Mar 8;19(3):e0299463. doi: 10.1371/journal.pone.0299463 (PMC10923479; doi:10.1371/journal.pone.0299463)
Supplement: S2 Fig — Proportions of 21 wader and 17 waterfowl species of populations during spring and autumn migration according to their population sizes and citizen science migration schedules. The most abundant species are labelled. (PDF) [file pone.0299463.s002.pdf]

Supporting Information S2 Fig

S2a

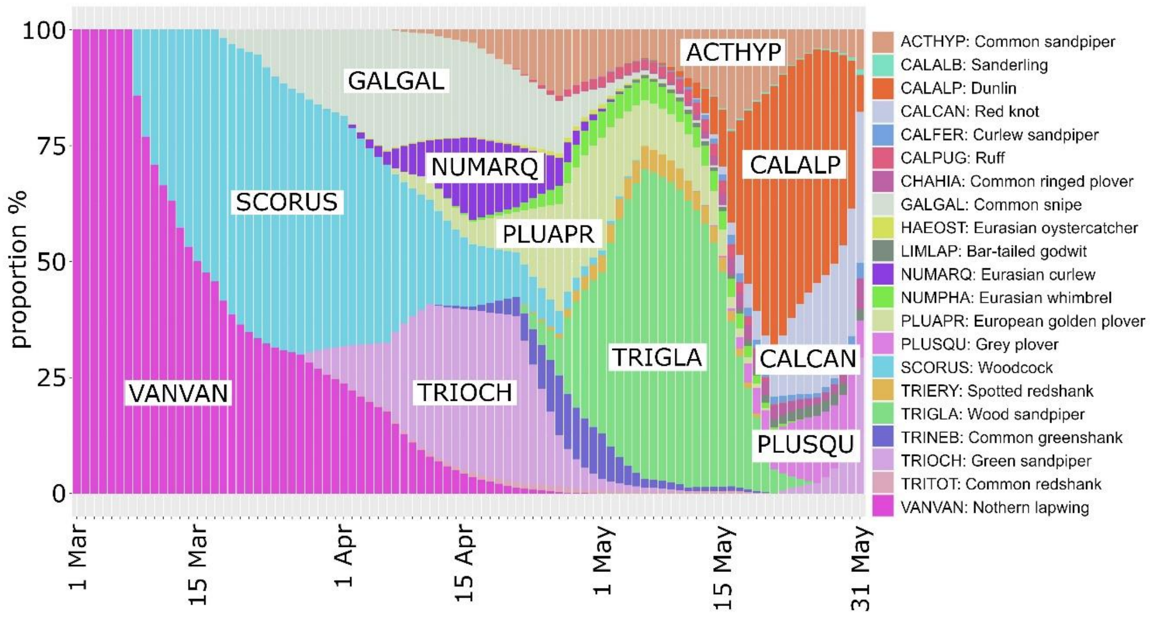

S2b

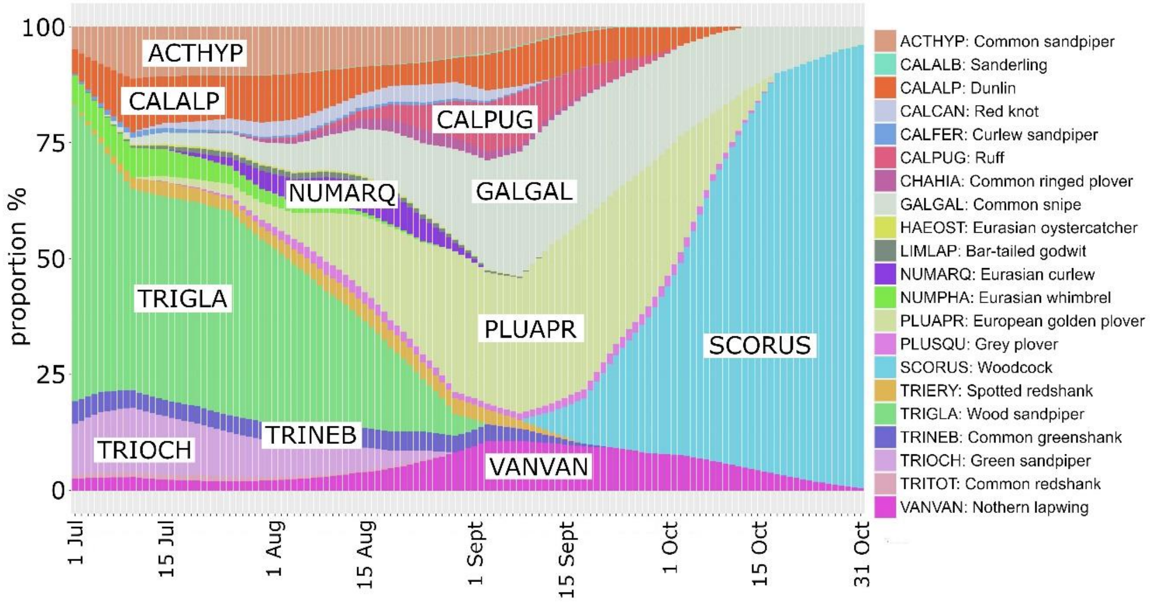

S2a and S2b Figs. Proportion of wader species in spring (a) and autumn (b) migration. Proportions of 21 wader species of wader populations during spring (a) and autumn (b) migration according to their population sizes and citizen science migration schedules. The most abundant species are labelled.

S2c

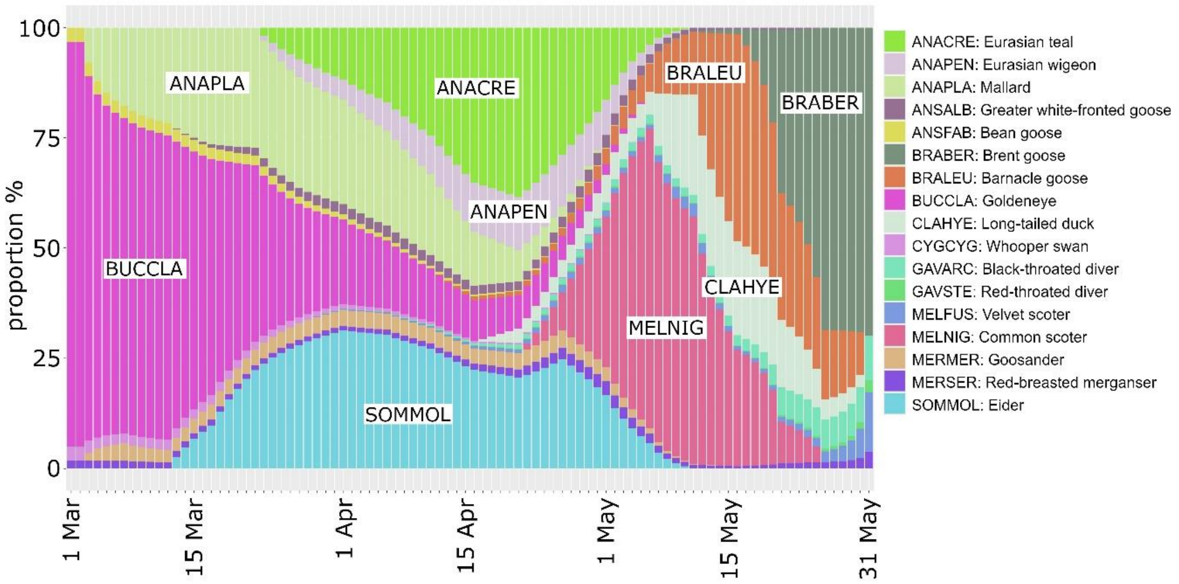

S2d

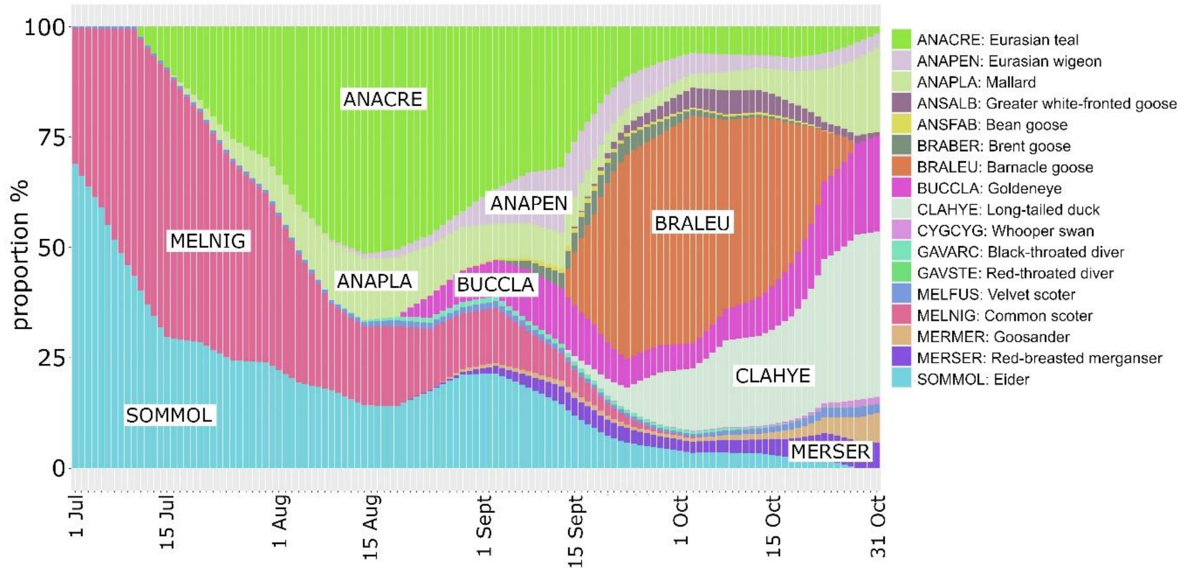

S2c and S2d Figs. Proportion of waterfowl species in spring (c) and autumn (d) migration. Proportion of 17 waterfowl species during spring (c) and autumn (d) migration according to their population sizes and citizen science migration schedules. The most abundant species are labelled.
