# Supplementary figures and images for: Evaluating the potential of bioacoustics in avian migration research by citizen science and weather radar observations
Source: PLoS One. 2024 Mar 8;19(3):e0299463. doi: 10.1371/journal.pone.0299463 (PMC10923479; doi:10.1371/journal.pone.0299463)

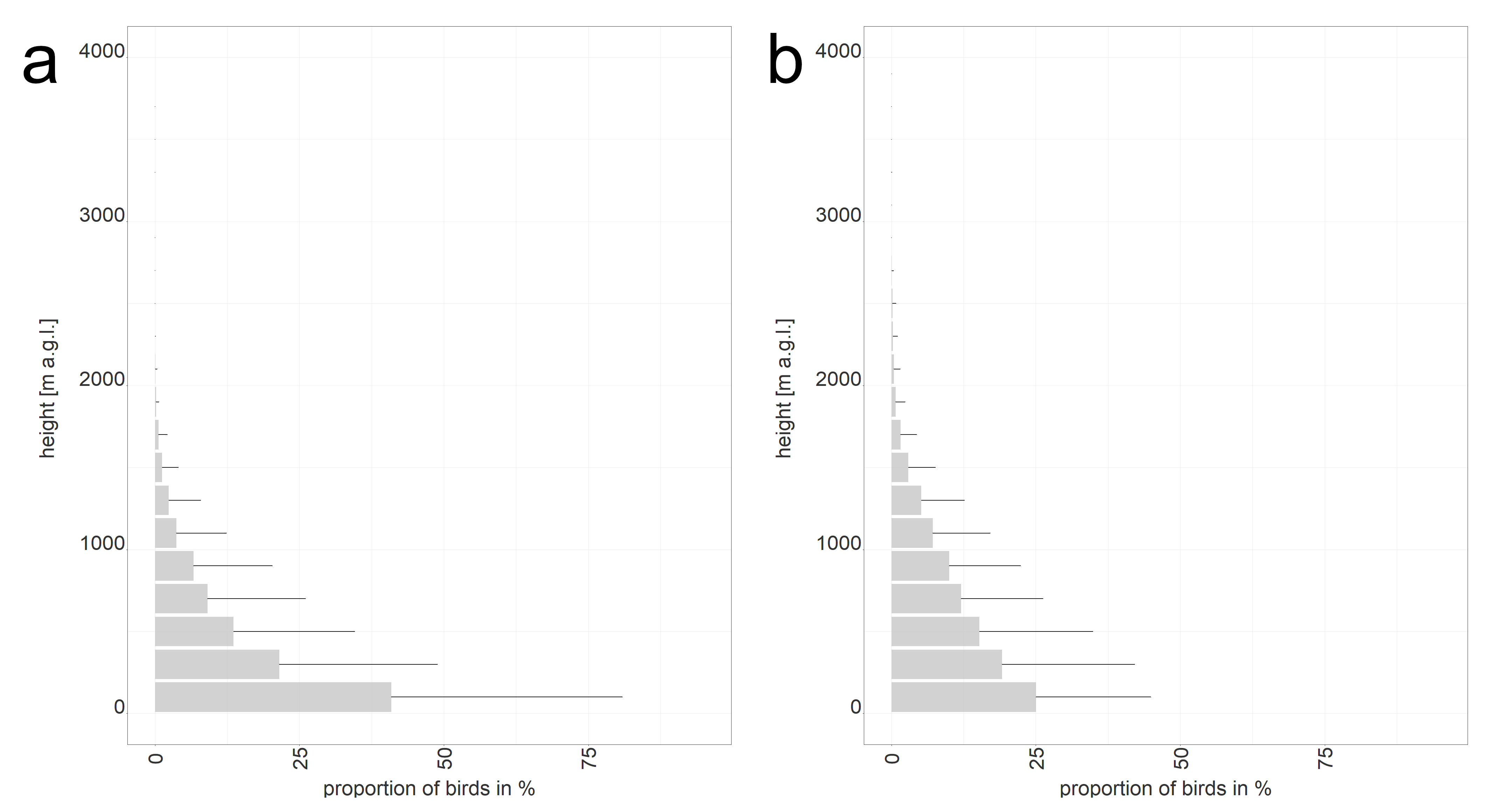

Supplement: S3 Fig — Mean proportions (and standard deviations) of birds (%) in 200-m height layers in the weather radar in Vihti, Finland, in spring (a) and autumn (b) 2022. (TIF) [file pone.0299463.s003.tif]
