# Supplementary material for: Evaluating the potential of bioacoustics in avian migration research by citizen science and weather radar observations
Source: PLoS One. 2024 Mar 8;19(3):e0299463. doi: 10.1371/journal.pone.0299463 (PMC10923479; doi:10.1371/journal.pone.0299463)
Supplement: S1 Table — Spring population size: estimated migrant population sizes in Southern Finland in spring; autumn population size: estimated migrant population sizes in Southern Finland in autumn; CS data: availability of citizen science migration schedules; Number of calling individuals in acoustic data: number of calling individuals in acoustic data from 2019–2022 for spring, autumn and in total. (PDF) [file pone.0299463.s004.pdf]

Species	spring population size	autumn population size	CS data	Number of calling individuals in acoustic data		
				spring	autumn	total
**Sparrowhawk (*Accipiter nisus* ; ACCNIS)**	19000	42000	x	0	0	0
**Blyth's reed warbler (*Acrocephalus dumetorum* ; ACRDUM)**	151000	502000	x	0	0	0
**Marsh warbler (*Acrocephalus palustris* ; ACRRIS)**	77000	209000	x	0	0	0
**Sedge warbler (*Acrocephalus schoenobaenus* ; ACRSCH)**	292000	836000	x	0	0	0
**Reed warbler (*Acrocephalus scirpaceus* ; ACRSCI)**	31000	83000	x	0	0	0
**Common sandpiper (*Actitis hypoleucos* ; ACTHYP)**	364000	556000	x	197	81	278
**Eurasian skylark (*Alauda arvensis* ; ALAARV)**	522000	1189000	x	27	36	63
**Northern pintail (*Anas acuta* ; ANAACU)**	92000	122000	x	0	0	0
**Northern shoveler (*Anas clypeata* ; ANACLY)**	34000	77000	x	0	0	0
**Eurasian teal (*Anas crecca* ; ANACRE)**	367000	937000	x	213	7	220
**Eurasian wigeon (*Anas penelope* ; ANAPEN)**	135000	261000	x	73	5	78
**Mallard (*Anas platyrhynchos* ; ANAPLA)**	250000	452000	x	30	22	52
**Gadwall (*Anas strepera* ; ANASTR)**				1	0	1
**Greater white-fronted goose (*Anser albifrons* ; ANSALB)**	50000	119000	x	80	1219	1299
**Grey-lag goose (*Anser anser* ; ANSANS)**			x	0	0	0
**Taiga bean goose (*Anser fabalis fabalis* ; ANSFABFAB)**	15000	23000	x	48	480	528
**Tundra bean goose (*Anser fabalis rossicus* ; ANSFABROS)**	25000	12000	x			
**Red-throated pipit (*Anthus cervinus* , ANTCER)**				0	1	1
**Meadow pipit (*Anthus pratensis* ; ANTPRA)**	2975000	7457000	x	0	8	8
**Tree pipit (*Anthus trivialis* ; ANTTRI)**	4915000	12334000	x	17	1065	1082
**Common swift (*Apus apus* ; APUAPU)**	36000	56000	x	1	1	2
**Grey heron (*Ardea cinerea* ; ARDCIN)**				19	61	80
**Tufted duck (*Aythya fuligula* ; AYTFUL)**	66000	128000	x	0	0	0
**Waxwing (*Bombycilla garrulus* ; BOMGAR)**	356000	681000	x	0	1	1

Species	spring population size	autumn population size	CS data	Number of calling individuals in acoustic data		
				spring	autumn	total
Eurasian bittern (*Botaurus stellaris* ; BOTSTE)				21	60	81
Brent goose (*Branta bernicla* ; BRABER)	200000	75000	x	0	40	40
Canada goose (*Branta canadensis* ; BRACAN)			x	60	20	80
Barnacle goose (*Branta leucopsis* ; BRALEU)	620000	1349000	x	5880	9779	15659
Goldeneye (*Bucephala clangula* ; BUCCLA)	288000	478000	x	325	68	393
Common buzzard (*Buteo buteo* ; BUTBUT)	15000	21000	x	0	0	0
Rough-legged buzzard (*Buteo lagopus* ; BUTLAG)	23000	38000	x	0	0	0
Sanderling (*Calidris alba* ; CALALB)	5000	8000	x	0	0	0
Dunlin (*Calidris alpina* ; CALALP)	372000	564000	x	3	5	8
Red knot (*Calidris canuta* ; CALCAN)	150000	139000	x	0	0	0
Broad-billed sandpiper (*Calidris falcinellus* ; CALFAL)	63000	102000	x	0	0	0
Curlew sandpiper (*Calidris ferruginea* ; CALFER)	20000	38000	x	0	0	0
Lapland bunting (*Calcarius lapponicus* ; CALLAP)				1	21	22
Little stint (*Calidris minuta* ; CALUTA)	10000	16000	x	0	0	0
Ruff (*Calidris pugnax* ; CALPUG)	59000	126000	x	0	0	0
Temminck's stint (*Calidris temminckii* , CALTEM)				0	1	1
European nightjar (*Caprimulgus europaeus* ; CAPEUR)				2	6	8
Linnet (*Carduelis cannabina* ; CARCAN)	15000	41000	x	0	0	0
Greenfinch (*Carduelis chloris* ; CARCHL)	0	210000		1	1	2
Common rosefinch (*Carpodacus erythrinus* ; CARERY)	273000	664000	x	0	0	0
Common redpoll (*Carduelis flammea* ; CARMEA)				1	153	154
Eurasian siskin (*Carduelis spinus* ; CARSPI)	3853000	9379000	x	0	28	28
Little ringed plover (*Charadrius dubius* ; CHADUB)				7	9	16
Common ringed plover (*Charadrius hiaticula* ; CHAHIA)	40000	56000	x	4	17	21
Long-tailed duck (*Clangula hyemalis* ; CLAHYE)	400000	626000	x	817	21	838
Wood pigeon (*Columba palumbus* ; COLPAL)	493000	900000	x	0	0	0

Species	spring population size	autumn population size	CS data	Number of calling individuals in acoustic data		
				spring	autumn	total
**Hooded crow (*Corvus cornix* ; CORNIX)**	195000	369000	x	1	0	1
**Jackdaw (*Corvus monedula* ; CORMON)**	52000	93000	x	2	0	2
**Corncrake (*Crex crex* ; CRECRE)**				2	0	2
**Cuckoo (*Cuculus canorus* ; CUCCAN)**	322000	705000	x	0	0	0
**Blue tit (*Cyanistes caeruleus* ; CYACAE)**	127000	381000	x	0	0	0
**Whooper swan (*Cygnus cygnus* ; CYGCYG)**	13000	24000	x	9	6	15
**Mute swan (*Cygnus olor* ; CYGOLO)**				0	1	1
**House martin (*Delichon urbica* ; DELURB)**	145000	354000	x	0	0	0
**Yellowhammer (*Emberiza citrinella* ; EMBCIT)**	284000	512000	x	18	133	151
**Ortolan bunting (*Emberiza hortulana* ; EMBHOR)**				1	3	4
**Reed bunting (*Emberiza schoeniclus* ; EMBSCH)**	1433000	3108000	x	3	33	36
**European robin (*Erithacus rubecula* ; ERIRUB)**	5225000	18009000	x	17	500	517
**Common kestrel (*Falco tinnunculus* ; FALTIN)**	17000	37000	x	0	0	0
**Pied flycatcher (*Ficedula hypoleuca* ; FICHYP)**	1580000	4694000	x	1	22	23
**Common chaffinch (*Fringilla coelebs* ; FRICOE)**	17194000	45602000	x	0	16	16
**Brambling (Fringilla montifringilla; FRIMON)**	7984000	22305000	x	12	191	203
**Coot (*Fulica atra* ; FULATR)**				52	2	54
**Moorhen (*Gallinula chloropus* ; GALCHL)**				50	2	52
**Common snipe (Gallinago gallinago; GALGAL)**	331000	658000	x	16	39	55
**Black-throated diver (*Gavia arctica* ; GAVARC)**	71000	27000	x	0	0	0
**Red-throated diver (*Gavia stellata* ; GAVSTE)**	7000	4000	x	0	1	1
**Common crane (*Grus grus* ; GRUGRU)**	65000	81000	x	10	65	75
**Oystercatcher (*Haematopus ostralegus* ; HAEOST)**	15000	18000	x	11	3	14
**Icterine warbler (*Hippolais icterina* ; HIPICT)**	60000	157000	x	0	0	0
**Barn swallow (*Hirundo rustica* ; HIRRUS)**	286000	730000	x	0	0	0
**Red-backed shrike (*Lanius collurio* ; LANCOL)**	110000	255000	x	0	0	0

Species	spring population size	autumn population size	CS data	Number of calling individuals in acoustic data		
				spring	autumn	total
Herring gull (Larus argentatus; LARARG)	86000	111000	x	1	0	1
Common gull (*Larus canus* ; LARCAN)	175000	260000	x	1	0	1
Lesser Black-backed gull (*Larus fuscus* ; LARFUS)	22000	29000	x	0	0	0
Little gull (*Hydrocoloeus minutus* , HYDMIN)				1	0	1
Black-headed gull (*Chroicocephalus ridibundus* ; CHRRID)	234000	396000	x	46	0	46
Bar-tailed godwit (*Limosa lapponica* ; LIMLAP)	29000	55000	x	20	4	24
Wood lark (*Lullula arborea* ; LULARB)				1	1	2
Thrush nightingale (*Luscinia luscinia* ; LUSLUS)	51000	138000	x	0	0	0
Bluethroat (*Luscinia svecica* ; LUSSVE)	384000	1089000	x	0	0	0
Velvet scoter (*Melanitta fusca* ; MELFUS)	46000	57000	x	0	0	0
Common scoter (*Melanitta nigra* ; MELNIG)	800000	501000	x	3873	10	3883
Goosander (*Mergus merganser* ; MERMER)	64000	171000	x	0	0	0
Red-breasted merganser (*Mergus serrator* ; MERSER)	48000	144000	x	0	0	0
White wagtail (*Motacilla alba* ; MOTALB)	905000	2258000	x	1	5	6
Yellow wagtail (*Motacilla flava* ; MOTFLA)	2138000	4883000	x	0	11	11
Spotted flycatcher (*Muscicapa striata* ; MUSSTR)	5158000	13363000	x	0	72	72
Eurasian curlew (*Numenius arquata* ; NUMARQ)	151000	174000	x	21	13	34
Eurasian whimbrel (*Numenius phaeopus* ; NUMPHA)	138000	199000	x	7	7	14
Wheatear (*Oenanthe oenanthe* ; OENOEN)	508000	1436000	x	0	0	0
Great tit (*Parus major* ; PARMAJ)	288000	643000	x	0	0	0
Honey buzzard (*Pernis apivorus* ; PERAPI)	6000	8000	x	0	0	0
Coal tit (*Periparus ater* ; PERATE)	16000	58000	x	0	0	0
Great cormorant (*Phalacrocorax carbo* ; PHACAR)	60000	96000	x	0	0	0
Common redstart (*Phoenicurus phoenicurus* ; PHOPHO)	2628000	9127000	x	2	0	2
Chiffchaff (*Phylloscopus collybita* ; PHYCOL)	621000	1997000	x	0	0	0

Species	spring population size	autumn population size	CS data	Number of calling individuals in acoustic data		
				spring	autumn	total
**Wood warbler (*Phylloscopus sibilatrix* ; PHYSIB)**	466000	1241000	x	0	0	0
**Greenish warbler (*Phylloscopus trochiloides* ; PHYDES)**	79000	166000		0	0	0
**Willow warbler (*Phylloscopus trochilus* ; PHYLUS)**	21133000	63631000	x	0	0	0
**Snow bunting (*Plectrophenax nivalis* ; PLENIV)**				10	16	26
**European golden plover (*Pluvialis apricaria* ; PLUAPR)**	463000	771000	x	14	12	26
**Grey plover (*Pluvialis squatarola* ; PLUSQU)**	80000	94000	x	0	10	10
**Great crested grebe (*Podiceps cristatus* ; PODCRI)**	91000	141000	x	0	0	0
**Spotted crake (*Porzana porzana* ; PORPOR)**				4	1	5
**Dunnock (*Prunella modularis* ; PRUMOD)**	961000	2020000	x	0	48	48
**Eurasian bullfinch (*Pyrrhula pyrrhula* ; PYRPYR)**				0	96	96
**Water rail (*Rallus aquaticus* ; RALAQU)**				45	9	54
**Goldcrest (*Regulus regulus* ; REGREG)**	1380000	6107000	x	2	92	94
**Sand martin (*Riparia riparia* ; RIPRIP)**	125000	342000	x	0	0	0
**Whinchat (*Saxicola rubetra* ; SAXTRA)**	537000	1370000	x	0	0	0
**Eurasian woodcock (*Scolopax rusticola* ; SCORUS)**	366000	736000	x	1	1	2
**Eider (*Somateria mollissima* ; SOMMOL)**	406000	619000	x	0	0	0
**Common tern (*Sterna hirundo* ; STEHIR)**	132000	200000	x	0	0	0
**Arctic tern (*Sterna paradisaea* ; STEAEA)**	121000	166000	x	0	0	0
**Starling (*Sturnus vulgaris* ; STUVUL)**	106000	236000	x	0	0	0
**Blackcap (*Sylvia atricapilla* ; SYLATR)**	234000	613000	x	0	0	0
**Garden warbler (*Sylvia borin* ; SYLBOR)**	1789000	4807000	x	0	0	0
**Common whitethroat (*Sylvia communis* ; SYLCOM)**	558000	1634000	x	0	0	0
**Lesser whitethroat (*Sylvia curruca* ; SYLCUR)**	549000	1524000	x	0	0	0
**Little grebe (*Tachybaptus ruficollis* ; TACRUF)**	0			0	1	1
**Spotted redshank (*Tringa erythropus* ; TRIERY)**	106000	177000	x	3	1	4
**Wood sandpiper (*Tringa glareola* ; TRIGLA)**	1361000	2374000	x	74	58	132

Species	spring population size	autumn population size	CS data	Number of calling individuals in acoustic data		
				spring	autumn	total
**Common greenshank (*Tringa nebularia* ; TRINEB)**	154000	257000	x	15	2	17
**Green sandpiper (*Tringa ochropus* ; TRIOCH)**	414000	638000	x	34	24	58
**Common redshank (*Tringa totanus* ; TRITOT)**	25000	45000	x	2	0	2
**Eurasian wren (*Troglodytes troglodytes* ; TROTRO)**	258000	689000	x	0	0	0
**Redwing (*Turdus iliacus* ; TURILI)**	3819000	8524000	x	789	4896	5685
**Common blackbird (*Turdus merula* ; TURMER)**	495000	1140000	x	206	514	720
**Song thrush (*Turdus philomelos* ; TURPHI)**	2460000	5034000	x	1002	3819	4821
**Fieldfare (*Turdus pilaris* ; TURPIL)**	2785000	6762000	x	184	281	465
**Mistle thrush (*Turdus viscivorus* ; TURVIS)**	589000	1212000	x	3	4	7
**Northern lapwing (*Vanellus vanellus* ; VANVAN)**	155000	305000	x	13	9	22
