# Supplementary material for: Evaluating the potential of bioacoustics in avian migration research by citizen science and weather radar observations
Source: PLoS One. 2024 Mar 8;19(3):e0299463. doi: 10.1371/journal.pone.0299463 (PMC10923479; doi:10.1371/journal.pone.0299463)
Supplement: S2 Table — Acoustic data consist of the four-year dataset from 2019–2022 and species with at least 10 call nights per season were included. For species which did not fulfil this criterion fields are marked as “NA”. (PDF) [file pone.0299463.s005.pdf]

Species	Spring						Autumn					
	Spearman		Linear Regression				Spearman		Linear Regression			
	rho	p	F	R squared	p	number of call nights	rho	p	F	R squared	p	number of call nights
**Common sandpiper (*Actitis hypoleucos*)**	0.84	<0.001	127.46	0.59	<0.001	32	0.63	<0.001	40.89	0.25	<0.001	44
**Eurasian skylark (*Alauda arvensis*)**	0.44	<0.001	21.86	0.20	<0.001	22	0.32	<0.001	1.68	0.01	0.20	17
**Eurasian teal (*Anas crecca*)**	0.69	<0.001	38.94	0.30	<0.001	36	NA	NA	NA	NA	NA	5
**Eurasian wigeon (*Anas penelope*)**	0.5	<0.001	16.45	0.15	<0.01	20	NA	NA	NA	NA	NA	2
**Mallard (*Anas platyrhynchos*)**	0.5	<0.001	26.51	0.23	<0.001	18	0.28	0.00	9.00	0.07	<0.01	11
**Greater white-fronted goose (*Anser albifrons*)**	NA	NA	NA	NA	NA	8	0.6	<0.001	28.67	0.19	<0.001	16
**Taiga/Tundra bean goose (*Anser fabalis*)**	NA	NA	NA	NA	NA	8	0.45	<0.001	13.33	0.10	<0.001	14
**Meadow pipit (*Anthus pratensis*)**	NA	NA	NA	NA	NA	0	NA	NA	NA	NA	NA	7
**Tree pipit (*Anthus trivialis*)**	0.32	0.00	8.33	0.08	<0.01	12	0.75	<0.001	43.13	0.26	<0.001	56
**Common swift (*Apus apus*)**	NA	NA	NA	NA	NA	1	NA	NA	NA	NA	NA	1
**Waxwing (*Bombycilla garrulus*)**	NA	NA	NA	NA	NA	0	NA	NA	NA	NA	NA	1
**Brent goose (*Branta bernicla*)**	NA	NA	NA	NA	NA	0	NA	NA	NA	NA	NA	1
**Canada goose (*Branta canadensis*)**	NA	NA	NA	NA	NA	7	NA	NA	NA	NA	NA	4
**Barnacle goose (*Branta leucopsis*)**	0.68	<0.001	8.76	0.09	<0.01	44	0.69	<0.001	35.51	0.23	<0.001	39
**Goldeneye (*Bucephala clangula*)**	0.29	0.00	9.72	0.10	<0.01	63	0.5	<0.001	25.18	0.17	<0.001	22
**Dunlin (*Calidris alpina*)**	NA	NA	NA	NA	NA	3	NA	NA	NA	NA	NA	5
**Eurasian siskin (*Carduelis spinus*)**	NA	NA	NA	NA	NA	0	-0.03	0.77	1.60	0.01	0.21	14
**Common ringed plover (*Charadrius hiaticula*)**	NA	NA	NA	NA	NA	4	NA	NA	NA	NA	NA	8
**Long-tailed duck (*Clangula hyemalis*)**	0.69	<0.001	46.69	0.34	<0.001	16	NA	NA	NA	NA	NA	2
**Hooded crow (*Corvus cornix*)**	NA	NA	NA	NA	NA	2	NA	NA	NA	NA	NA	0
**Jackdaw (*Corvus monedula*)**	NA	NA	NA	NA	NA	1	NA	NA	NA	NA	NA	0
**Whooper swan (*Cygnus cygnus*)**	NA	NA	NA	NA	NA	5	NA	NA	NA	NA	NA	4
**Yellowhammer (*Emberiza citrinella*)**	0.22	0.03	6.31	0.07	0.01	13	0.75	<0.001	64.73	0.35	<0.001	24
**Reed bunting (*Emberiza schoeniclus*)**	NA	NA	NA	NA	NA	3	0.53	<0.001	22.13	0.15	<0.001	18
**European robin (*Erithacus rubecula*)**	0.21	0.05	2.36	0.03	0.13	14	0.76	<0.001	65.30	0.35	<0.001	59
**Pied flycatcher (*Ficedula hypoleuca*)**	NA	NA	NA	NA	NA	1	0.46	<0.001	29.46	0.20	<0.001	13
**Common chaffinch (*Fringilla coelebs*)**	NA	NA	NA	NA	NA	0	0.28	0.00	15.67	0.11	<0.001	13
**Brambling (*Fringilla montifringilla*)**	NA	NA	NA	NA	NA	3	0.74	<0.001	18.51	0.13	<0.001	34
**Common snipe (*Gallinago gallinago*)**	0.29	0.01	9.77	0.10	<0.01	11	0.21	0.02	3.12	0.03	0.08	24
**Red-throated diver (*Gavia stellata*)**	NA	NA	NA	NA	NA	0	NA	NA	NA	NA	NA	1
**Common crane (*Grus grus*)**	NA	NA	NA	NA	NA	1	NA	NA	NA	NA	NA	4
**Oystercatcher (*Haematopus ostralegus*)**	NA	NA	NA	NA	NA	9	NA	NA	NA	NA	NA	3
**Herring gull (*Larus argentatus*)**	NA	NA	NA	NA	NA	1	NA	NA	NA	NA	NA	0
**Common gull (*Larus canus*)**	NA	NA	NA	NA	NA	1	NA	NA	NA	NA	NA	0
**Black-headed gull (*Chroicocephalus ridibundus*)**	0.46	<0.001	22.31	0.20	<0.001	19	NA	NA	NA	NA	NA	0

Species	Spring						Autumn					
	Spearman		Linear Regression				Spearman		Linear Regression			
	rho	p	F	R squared	p	number of call nights	rho	p	F	R squared	p	number of call nights
**Bar-tailed godwit (*Limosa lapponica*)**	NA	NA	NA	NA	NA	2	NA	NA	NA	NA	NA	2
**Common scoter (*Melanitta nigra*)**	0.71	<0.001	14.21	0.14	<0.001	22	NA	NA	NA	NA	NA	2
**White wagtail (*Motacilla alba*)**	NA	NA	NA	NA	NA	1	NA	NA	NA	NA	NA	5
**Yellow wagtail (*Motacilla flava*)**	NA	NA	NA	NA	NA	0	NA	NA	NA	NA	NA	4
**Spotted flycatcher (*Muscicapa striata*)**	NA	NA	NA	NA	NA	0	0.6	<0.001	36.12	0.23	<0.001	24
**Eurasian curlew (*Numenius arquata*)**	0.41	<0.001	13.93	0.13	<0.001	11	NA	NA	NA	NA	NA	9
**Eurasian whimbrel (*Numenius phaeopus*)**	NA	NA	NA	NA	NA	5	NA	NA	NA	NA	NA	4
**Common redstart (*Phoenicurus phoenicurus*)**	NA	NA	NA	NA	NA	2	NA	NA	NA	NA	NA	0
**European golden plover (*Pluvialis apricaria*)**	0.48	<0.001	25.29	0.22	<0.001	10	0.25	0.01	7.31	0.06	0.01	12
**Grey plover (*Pluvialis squatarola*)**	NA	NA	NA	NA	NA	0	NA	NA	NA	NA	NA	7
**Dunnock (*Prunella modularis*)**	NA	NA	NA	NA	NA	0	0.6	<0.001	67.65	0.36	<0.001	22
**Goldcrest (*Regulus regulus*)**	NA	NA	NA	NA	NA	2	0.72	<0.001	50.62	0.30	<0.001	30
**Eurasian woodcock (*Scolopax rusticola*)**	NA	NA	NA	NA	NA	1	NA	NA	NA	NA	NA	1
**Spotted redshank (*Tringa erythropus*)**	NA	NA	NA	NA	NA	2	NA	NA	NA	NA	NA	1
**Wood sandpiper (*Tringa glareola*)**	0.54	<0.001	3.47	0.04	0.07	12	0.62	<0.001	33.22	0.22	<0.001	29
**Common greenshank (*Tringa nebularia*)**	NA	NA	NA	NA	NA	7	NA	NA	NA	NA	NA	2
**Green sandpiper (*Tringa ochropus*)**	0.49	<0.001	11.26	0.11	<0.01	19	0.48	<0.001	37.63	0.24	<0.001	22
**Common redshank (*Tringa totanus*)**	NA	NA	NA	NA	NA	2	NA	NA	NA	NA	NA	0
**Redwing (*Turdus iliacus*)**	0.71	<0.001	14.94	0.14	<0.001	37	0.8	<0.001	30.12	0.20	<0.001	83
**Common blackbird (*Turdus merula*)**	0.82	<0.001	80.66	0.47	<0.001	43	0.87	<0.001	69.10	0.36	<0.001	60
**Song thrush (*Turdus philomelos*)**	0.87	<0.001	60.62	0.40	<0.001	50	0.88	<0.001	148.47	0.55	<0.001	84
**Fieldfare (*Turdus pilaris*)**	0.65	<0.001	1.71	0.02	0.19	27	0.88	<0.001	106.77	0.47	<0.001	47
**Mistle thrush (*Turdus viscivorus*)**	NA	NA	NA	NA	NA	2	NA	NA	NA	NA	NA	3
**Northern lapwing (*Vanellus vanellus*)**	NA	NA	NA	NA	NA	9	NA	NA	NA	NA	NA	9
