# Supplementary material for: Evaluating the potential of bioacoustics in avian migration research by citizen science and weather radar observations
Source: PLoS One. 2024 Mar 8;19(3):e0299463. doi: 10.1371/journal.pone.0299463 (PMC10923479; doi:10.1371/journal.pone.0299463)
Supplement: S3 Table — Species with at least 10 call nights per season were included. For species which did not fulfil this criterion percentiles are labelled as “NA”. (PDF) [file pone.0299463.s006.pdf]

Species	Spring 5%	Spring 50%	Spring 95%	Autumn 5%	Autumn 50%	Autumn 95%
**Common sandpiper (*Actitis hypoleucos*)**	26-Apr	6-May	19-May	14-Jul	9-Aug	28-Aug
**Eurasian skylark (*Alauda arvensis*)**	10-Mar	27-Mar	5-May	NA	NA	NA
**Eurasian teal (*Anas crecca*)**	6-Apr	25-Apr	5-May	NA	NA	NA
**Eurasian wigeon (*Anas penelope*)**	25-Mar	22-Apr	5-May	NA	NA	NA
**Mallard (*Anas platyrhynchos*)**	24-Mar	8-Apr	20-Apr	18-Sep	20-Oct	26-Oct
**Tree pipit (*Anthus trivialis*)**	8-Apr	27-Apr	28-May	18-Aug	1-Sep	25-Sep
**Barnacle goose (*Branta leucopsis*)**	20-Apr	15-May	23-May	27-Sep	8-Oct	19-Oct
**Goldeneye (*Bucephala clangula*)**	18-Mar	11-Apr	19-May	15-Sep	12-Oct	30-Oct
**Eurasian siskin (*Carduelis spinus*)**	NA	NA	NA	14-Aug	19-Oct	31-Oct
**Long-tailed duck (*Clangula hyemalis*)**	8-May	17-May	19-May	NA	NA	NA
**Yellowhammer (*Emberiza citrinella*)**	2-Mar	2-Apr	13-May	9-Oct	21-Oct	30-Oct
**Reed bunting (*Emberiza schoeniclus*)**	NA	NA	NA	20-Sep	6-Oct	29-Oct
**European robin (*Erithacus rubecula*)**	6-Apr	2-May	28-May	2-Sep	27-Sep	16-Oct
**Pied flycatcher (*Ficedula hypoleuca*)**	NA	NA	NA	11-Aug	20-Aug	8-Sep
**Common chaffinch (*Fringilla coelebs*)**	NA	NA	NA	29-Jul	25-Sep	30-Oct
**Brambling (*Fringilla montifringilla*)**	NA	NA	NA	18-Sep	7-Oct	21-Oct
**Common snipe (*Gallinago gallinago*)**	6-Mar	27-Apr	4-May	14-Aug	10-Sep	24-Oct
**Black-headed gull (*Chroicocephalus ridibundus*)**	1-Apr	18-Apr	9-May	NA	NA	NA
**Common scoter (*Melanitta nigra*)**	30-Apr	6-May	12-May	NA	NA	NA
**Spotted flycatcher (*Muscicapa striata*)**	NA	NA	NA	10-Aug	22-Aug	10-Sep
**European golden plover (*Pluvialis apricaria*)**	12-Apr	3-May	13-May	3-Aug	31-Aug	13-Oct
**Dunnock (*Prunella modularis*)**	NA	NA	NA	9-Sep	24-Sep	9-Oct

Species	Spring 5%	Spring 50%	Spring 95%	Autumn 5%	Autumn 50%	Autumn 95%
**Goldcrest (*Regulus regulus*)**	NA	NA	NA	15-Sep	5-Oct	11-Oct
**Wood sandpiper (*Tringa glareola*)**	NA	NA	NA	20-Jul	6-Aug	28-Aug
**Green sandpiper (*Tringa ochropus*)**	11-Apr	26-Apr	27-May	6-Jul	28-Jul	21-Aug
**Redwing (*Turdus iliacus*)**	8-Apr	19-Apr	4-May	8-Sep	28-Sep	21-Oct
**Common blackbird (*Turdus merula*)**	12-Mar	27-Mar	19-Apr	18-Sep	12-Oct	29-Oct
**Song thrush (*Turdus philomelos*)**	12-Apr	26-Apr	8-May	4-Sep	26-Sep	12-Oct
**Fieldfare (*Turdus pilaris*)**	6-Apr	27-Apr	29-Apr	20-Sep	11-Oct	30-Oct
